# Differential gene expression induced by Verteporfin in endometrial cancer cells

**DOI:** 10.1038/s41598-019-40495-9

**Published:** 2019-03-07

**Authors:** Lisa Gahyun Bang, Venkata Ramesh Dasari, Dokyoon Kim, Radhika P. Gogoi

**Affiliations:** 1 Biomedical and Translational Informatics Institute, Geisinger, Danville, PA USA; 2 Weis Center for Research, Geisinger Clinic, Danville, PA USA; 3 Huck Institute of the Life Sciences, Pennsylvania State University, University Park, PA USA; 4 Geisinger Medical Center, Danville, PA USA

## Abstract

Endometrial cancer (EMCA) is a clinically heterogeneous disease. Previously, we tested the efficacy of Verteporfin (VP) in EMCA cells and observed cytotoxic and anti-proliferative effects. In this study, we analyzed RNA sequencing data to investigate the comprehensive transcriptomic landscape of VP treated Type 1 EMCA cell lines, including HEC-1-A and HEC-1-B. There were 549 genes with differential expression of two-fold or greater and P < 0.05 after false discovery rate correction for the HEC-1-B cell line. Positive regulation of *TGF*β1 production, regulation of lipoprotein metabolic process, cell adhesion, endodermal cell differentiation, formation and development, and integrin mediated signaling pathway were among the significantly associated terms. A functional enrichment analysis of differentially expressed genes after VP treatment revealed extracellular matrix organization Gene Ontology as the most significant. *CDC23* and *BUB1B*, two genes crucially involved in mitotic checkpoint progression, were found to be the pair with the best association from STRING among differentially expressed genes in VP treated HEC-1-B cells. Our *in vivo* results indicate that subcutaneous tumors in mice were regressed after VP treatment by inhibiting cell cycle pathway proteins. The present study revealed multiple key genes of pathological significance in EMCA, thereby improving our understanding of molecular profiles of EMCA cells.

## Introduction

Endometrial cancer (EMCA) is a clinically heterogeneous disease. Majority of endometrial carcinomas are generally low grade and low stage with favorable prognoses, however, the high-grade EMCA accounts for a disproportionate number of EMCA deaths^1–3^. EMCA has been grouped into 2 types. Type 1 is estrogen potentiated, estrogen receptor (ER) and progesterone receptor (PR) positive, and generally carries a favorable prognosis. Type 2 is ER/PR negative tumors, of non-endometrioid histology (mainly serous and clear cell carcinoma), are seen in post-menopausal women, and are associated with atrophic endometrium, and poor outcomes^1^. High-grade endometrial carcinoma constitutes a biologically, morphologically, genetically, and clinically heterogeneous group of tumors. Recent developments of large-scale genomic studies reveal that this heterogeneity may be a function of the diversity of various molecular alterations during disease progression. The analyses using The Cancer Genome Atlas (TCGA) data have led to an integrated genomic classification of endometrioid endometrial carcinomas (EECs) and serous endometrial carcinomas (SECs) and the identification of the *POLE* (ultramutated), microsatellite instability (MSI) (hypermutated), copy-number low (endometrioid) and copy-number high (serous-like) subtypes, with distinct combinations of genomic and epigenetic alterations^1^. Mounting evidence suggests that some molecular alterations are preferentially found in endometrioid endometrial carcinomas (EECs), including mutations in *PTEN* and *CTNNB1*, whereas others such as *TP53* mutations are more prevalent in serous endometrial carcinomas (SECs)^2,3^.

The American Cancer Society estimates that 63,230 new cases of cancer of the body of the uterus (uterine body or corpus) will be diagnosed and about 11,350 women will die from cancers of the uterine body^4^. Although there are many drugs approved for the treatment of ovarian cancer, there is only one FDA-approved drug (Megestrol Acetate) for EMCA, highlighting the need for new therapies to treat advanced, recurrent and metastatic EMCA^5,6^. Our laboratory identified nuclear expression of the Yes-associated protein (YAP) as a poor prognostic indicator in the overall survival of patients with EMCA^7^. YAP, the main downstream target of the Hippo pathway, plays an important role in the balance between cell proliferation and apoptosis^8–10^. Verteporfin (VP)^11^, an FDA approved drug used in photodynamic therapy (PDT) for adult macular degeneration was recently identified as an inhibitor of YAP and its binding partner TEA Domain Transcription Factor 1 (TEAD) binding^12^. Since the identification of VP as a YAP/TEAD inhibitor, several *in vitro* and *in vivo* studies have revealed the new potential of YAP1 in different cancers, where YAP is overexpressed^13–16^. We tested the efficacy of VP treatment in Type 1 EMCA cells (HEC-1-A and HEC-1-B) and observed cytotoxic and anti-proliferative effects^17^. Based on the molecular heterogeneity observed in EMCA patients and the effects of VP on EMCA cells, we hypothesized that VP might alter the biological processes and pathways associated with progression of EMCA cells. The aims of this study were to study the effects of VP on (1) genes or gene expression modules representative of biological processes known to play a role in EMCA, and (2) to define the association of these genes and/or pathways in the progression of EMCA, using RNA sequencing data. Here, we have used RNA sequencing (RNA-seq) to develop transcriptome data set of control and VP treated EMCA cells. We preferred RNA-seq compared to microarray technologies, as RNA-seq has a better dynamic range in estimates of gene expression and better precision^18^.

## Materials and Methods

### EMCA cell lines and culture conditions

We used Type 1 EMCA cells for the RNA-seq analysis portion of this study. HEC-1-A (ATCC, HTB-112) and HEC-1-B (ATCC, HTB-113) were obtained from the American Type Culture Collection (ATCC) (Manassas, VA). Both these cell lines were isolated from a patient with stage IA endometrial cancer. HEC-1-A cells were cultured in McCoy’s 5A medium (ATCC, Manassas, VA) supplemented with 10% (v/v) fetal bovine serum (FBS) (Thermo Fisher Scientific, Waltham, MA), HEC-1-B in Eagle’s minimum essential medium (EMEM) (ATCC, Manassas, VA) supplemented with 10%(v/v) FBS. Antibiotics (10 units/ml of penicillin and 10 mg/ml of streptomycin) were added to all culture media. Both cell lines were incubated at 37 °C in a humidified atmosphere containing 5% carbon dioxide.

### Verteporfin (VP) treatment

Verteporfin **(**Sigma, Cat. No. SML0534) was dissolved in DMSO and added to the medium for a final concentration of 10 nM and the cells were treated for 3 h. Equal concentration of DMSO was added to the control cells.

### Sample and library preparation

After VP treatment for 3 h at 10 nM, total RNA was isolated from EMCA cells using RNeasy Plus Mini Kit (Qiagen) with DNase treatment. The RNA concentration and purity were measured using the Nanodrop spectrophotometer (Thermo Fisher Scientific), and RNA integrity was evaluated with the Bioanalyzer RNA 6000 Nano Chip. RNA quality was uniformly excellent and met the following criteria; Nanodrop, 260/280 ratio >1.8; Bioanalyzer, RIN > 6.6. The samples were prepared using Illumina TruSeq Stranded Total RNA Sample Prep Kit with Ribo-Zero Gold and then subjected to 125 cycle paired-end sequencing. The average insert size of libraries constructed with the Illumina TruSeq Stranded Total RNA Sample Prep Kit is approximately 150 bp with inserts ranging from 100 to 400 bp. For the RNA-seq analysis, there were two replicates of the control samples (C) and VP-treated samples (VP) for each cell line (HEC-1-A and HEC-1-B) for a total of 8 samples. Sequencing and analysis were done by High-Throughput Genomics and Bioinformatic Analysis, University of Utah Shared Resources (https://healthcare.utah.edu/huntsmancancerinstitute/research/shared-resources/center-managed/bioinformatics/).

### Sequence reads alignment and transcript assembly

After processing on the Illumina HiSeq 2500 instrument, FASTQ files containing the nucleotide scores and quality scores for each position were generated; these reads were aligned to the human reference genome (Ensembl release 75) using the STAR read aligner^19^, specifying that the sequencing experiment was paired-end. The alignment step produced a SAM file. SAMtools was used to generate BAM files from these for the sequencing runs required to generate count matrices^20^. After reading in the genomic features model (Ensembl’s GTF file) to count reads/fragments and ensuring none of our sequences were circular, a count of all of the exons grouped by gene was calculated.

### Analysis of differentially expressed genes

**Figure 1:**
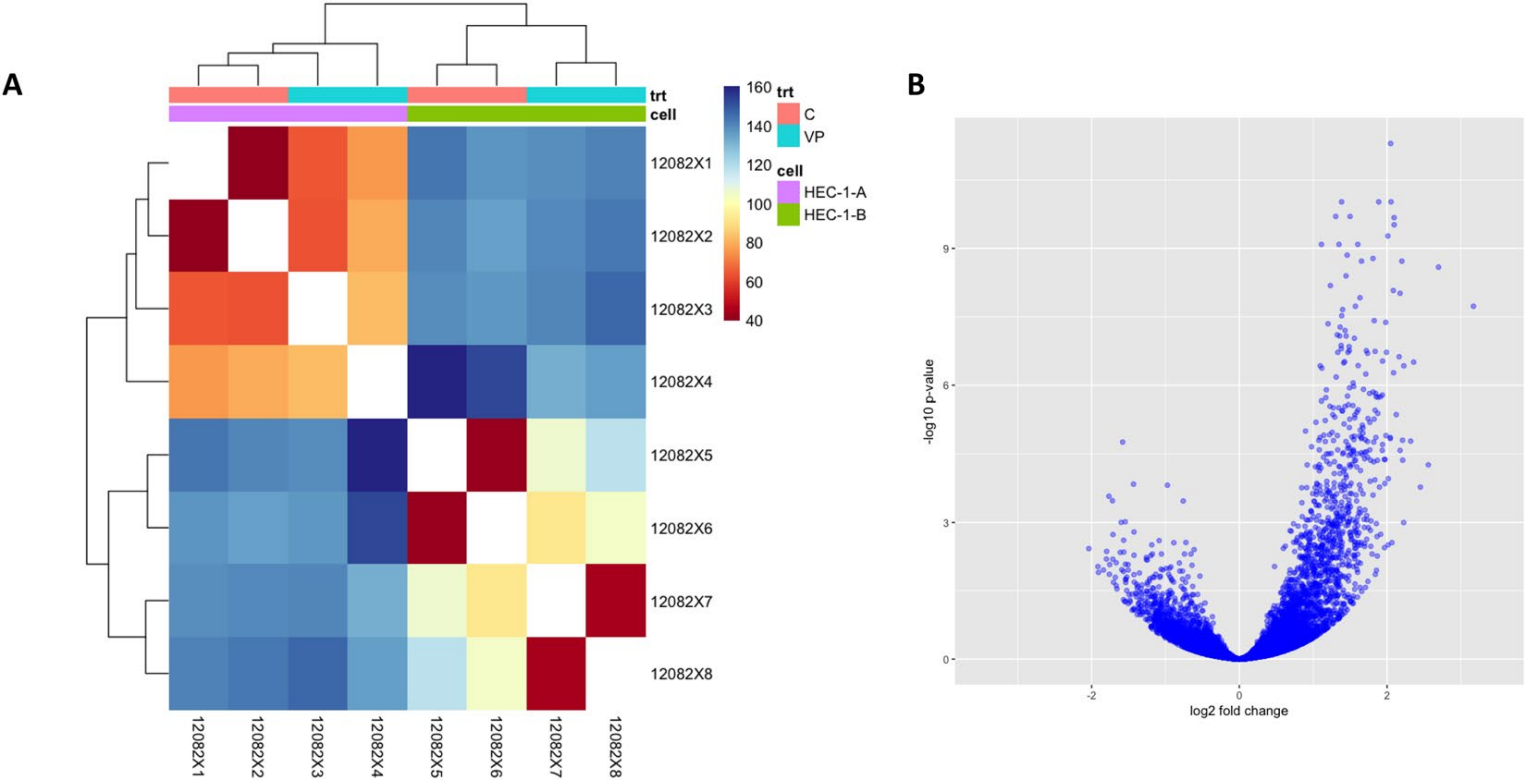
Gene expression modulated by VP. (**A**) Expression heat map of sample-to-sample distances on the matrix of variance-stabilized data for overall gene expression. trt = treatment; C = Control; VP = Verteporfin treated. Clustering differentiates between control and VP treated samples. This heatmap was built using DESeq 2 on normalized gene read counts. All of the rlog values of the dispersion estimates were clustered using the R distance function (*dist*) to calculate the Euclidean distance between samples. Distance plot for HEC-1A and HEC-1B cell lines showing their control and VP-treated versions. (**B**) Differential gene expression, with fold difference between log_2_ normalized expression in control (n = 2) and VP treated (n = 2) plotted versus −log_10_ adjusted P-value. Each gene is colored based on the log_10_ base mean expression.

The Bioconductor RNA-seq workflow was followed to detect differential expression^21^, using the DESeq 2 and other Bioconductor packages in R^22^. Because counts for transcripts in RNA-seq data can contain many rows with only zeros, rows with little to no information about the amount of gene expression were removed to reduce the size of the data object and to increase computational speed. When analyzing the general effect of VP treatment in both cell lines, standard RNA-seq workflow was followed in removing 292 features with zero reads and 1087 features with 5 of more reads. For the analysis using only HEC-1-B samples, we removed 656 features with zero counts and 1,776 features with fewer than 5 reads. The correlation plots between the replicates of the cell lines (e.g. 2 replicates of HEC-1-A control) show good correlation of normalized transcript counts (measured in log10(FPKM + 1)). DESeq 2 feature counts and other functions were run on the tables of counts to determine differentially expressed genes before and after VP-treatment. Results were considered statistically significant at an adjusted p < 0.05 (DMSO treated vs VP treated). The table of counts for each condition were used as inputs to the DESeq feature counts function to determine differentially expressed genes before and after VP-treatment. Since the expected variance of RNA-seq counts increases with the mean, DESeq 2’s regularized-logarithm transformation (rlog) of the count data was used to steady the variance across the mean^21,22^. The regularized log transforms (rlog) were taken of the result to generate a matrix of regularized counts for sample visualizations (Fig. 1). To check gene expression after VP treatment, the top 40 most significant genes were sorted by log fold change (logFC) and clustered in a hierarchical fashion using the rlog differences. This was plotted in volcano plots and heatmaps with accompanying dendrograms using pheatmap and ggplot2 packages in R^23^. An FDR-adjusted P < 0.05 was considered significant^24^.

To compare VP-treated and untreated samples, a logFC shrinkage method (apeglm) was used to get true low bias logFC estimates for true large differences^25^. This shrinkage method uses a Bayesian procedure to moderate fold changes from genes with very low and highly variable counts. This serves to reduce noise in differential transcript counts. The logFC after VP treatment was plotted on the y-axis and the average of the counts normalized by size factor plotted on the x-axis to create an MA-plot with no shrinkage and one with logFC shrinkage (Supplementary Fig. S3).

**Figure 2:**
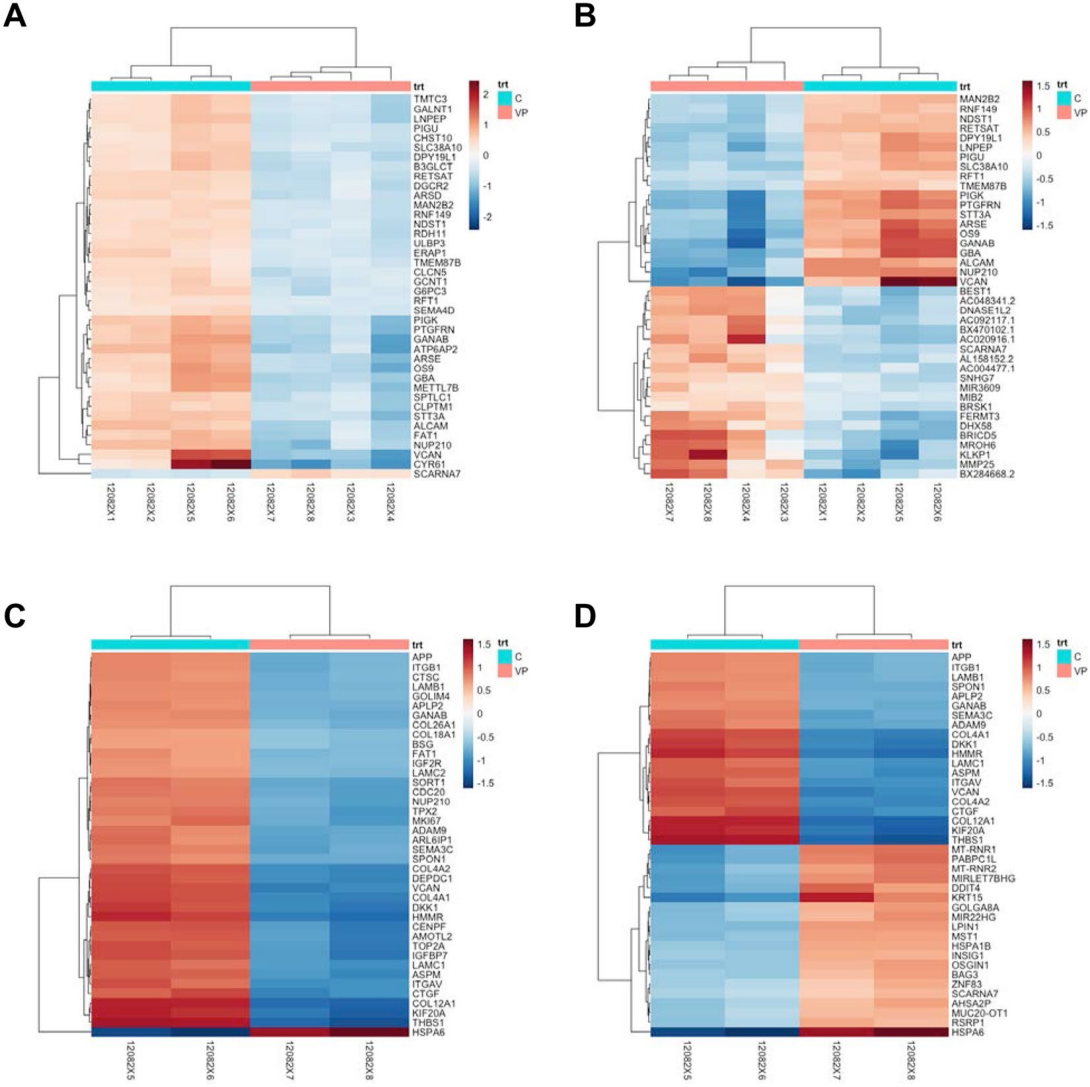
Differential RNA-seq feature counts for both EMCA cell lines. (**A**) Heatmap showing differential RNA-seq feature counts for both EMCA cell lines (HEC-1-A and HEC-1-B) before and after VP treatment sorted by logFC. Heatmap shows genes with RNA expression most altered after VP treatment. (**B**) Heatmap showing differential RNA-seq feature counts for both HEC-1A and HEC-1B EMCA cell lines combined, before and after VP treatment sorted by adjusted p-value, top 20 up and top 20 down-regulated genes are shown. (**C**) Differential RNA-seq feature counts for HEC-1-B cells before and after VP treatment sorted by logFC. Heatmap of genes with RNA expression most altered after VP treatment, sorted by log Fold Change (logFC). There were two replicates of the control samples (C) and VP-treated samples (VP) for 4 total HEC-1B samples. (**D**) Heatmap showing differential RNA-seq feature counts for HEC-1-B cells before and after VP treatment sorted by adjusted p-value, top 20 up and top 20 down-regulated genes.

The top 20 up- and down- regulated genes were also sorted by p-value (adjusted for multiple testing using FDR) and plotted those values in heatmaps as well using the pheatmap package wrapped inside the Huntsman Cancer Institute’s hciR package (Fig. 2). False discovery rates (FDR) were calculated by determining the number of control sample differential expression ratios (i.e. ratios of expression values between replicate controls) that exceeded an VP treated sample/control sample ratio and dividing by the rank order of the VP treated sample/control sample ratio. This is the FDR, i.e. fraction of genes that have higher apparent differential expression ratios by chance. 5% of genes with an FDR ≤ 0.05 are expected to be false discoveries. Only genes for which at least one condition had a differential expression ratio with an FDR ≤ 0.05 were included in further analysis. These were converted to log_2_ values.

### Protein-protein interaction network

To investigate the interactions between the protein products of top differentially expressed genes in the HEC-1-B cell line following VP treatment, a STRING network was constructed with the nodes consisting of genes and edges derived from experimentally validated protein-protein interactions. From the list of differentially expressed genes, a hypergeometric score was calculated of the likelihood of co-occurrence of genes (Supplementary Table S3). The Pfam protein domains and KEGG pathways most associated with the differentially expressed genes were also obtained through STRING (Supplementary Table S5). Many of the genes altered in HEC-1-B alone seemed to overlap with genes in the cell cycle pathway; a simple overlap test was performed to produce the list of cell cycle pathway genes differentially expressed between VP-treated and Control.

### shRNA treatment

The shRNA targeting the YAP gene was used for downregulating YAP. The control siRNA (sc-37007; Santa Cruz) was used as a negative control. shYAP was transfected into EMCA cells (70% confluency) using Lipofectamine3000 (Invitrogen) according to the manufacturer’s instructions. After 72 hours of transfection, the cells were harvested and used for checking the downregulation of YAP on cell cycle proteins. The knockdown of the YAP was verified by western blotting.

### Quantitative real time PCR

All primer sequences were determined using established human or mouse GenBank sequences. Primer sequences were designed using PrimerQuest (IDT) software (Supplementary Table S9). For Real-time polymerase chain reaction (RT-PCR) analysis, total RNA was isolated from control and VP (10 nM, 3 h) treated EMCA cells. Total cellular RNA was extracted with RNeasy kit (Qiagen) with on-column DNase treatment. We used RNA whose A_260_: A_280_ ratio is ≥2.0. Total RNA was reverse transcribed into first strand cDNA using iScript cDNA Synthesis Kit (Bio-Rad), as per the manufacturer’s instructions. Quantitative analysis of genes was done by SYBR green based real-time PCR using Applied Biosystems Real-Time PCR Detection System. Each sample was measured in triplicate and normalized to the reference GAPDH/β-actin/PGK1/LDHA/PPIH gene expression. A statistical evaluation of RT-PCR results was performed using one-way analysis of variance (ANOVA) to compare test gene expression between control and VP treated cells.

### Western blot analysis

Control and VP-treated mice tissues or control and VP-treated cells were lysed in RIPA buffer (Boston Bioproducts, Cat. No. BP-115DG) supplemented with protease and phosphatase inhibitors and subjected to SDS-PAGE. Samples were separated electrophoretically on 10% to 12% gels, electroblotted onto nitrocellulose membrane (Bio-Rad), blots were blocked at room temperature for 1 h in 5% (w/v) milk in phosphate-buffered saline and incubated overnight at 4 °C with primary antibodies. Protein bands were visualized with SuperSignal™ West Pico Chemiluminescent Substrate (Thermo Fisher Scientific) and detected using LAS-3000 (Fujifilm, Tokyo, Japan). All the antibodies used in this study and their details were provided in Supplementary Table S10.

### Mice experiments

To evaluate the efficacy of VP for inhibition of tumor growth in a mouse model, we injected HEC-1-B GFP cells into NCr nude mice by subcutaneous (SC) administration. Mice experiments were conducted by Anticancer Inc. (http://www.anticancer.com). All animal studies were conducted with an Anticancer Institutional Animal Care and Use Committee (IACUC)-protocol specifically approved for this study and in accordance with the principles and procedures outlined in the National Institute of Health Guide for the Care and Use of Animals. All animal procedures were carried out under specific-pathogen-free (SPF) conditions. Log-phase HEC-1-B GFP cells (5 × 10^6^) were suspended in 100 µl PBS and injected SC in left flank of mouse. After the tumors reached a size >100 mm^3^, VP was administered IP at a dose 50 mg/kg bodyweight to VP-treated group mice (n = 10). VP was given 3 times a week for 3 weeks. Control group mice (n = 10) were administered DMSO in a similar manner. Tumor size was measured by caliper and by GFP imaging twice a week. Body weight was measured twice per week and Body condition scores (BCS) were taken daily. Mice were sacrificed after 3 weeks of treatment. Primary tumors were excised and weighed at necropsy. Blood samples were collected, immediately processed to prepare plasma and flash frozen. Based on tumor volume, VP-treated mice were divided into Responders (tumor volume <500 mm^3^) and Non-responders (tumor volume >500 mm^3^). There were 6 mice in the Responders group (animal numbers 1, 2, 4, 5, 6 and 8) and 4 mice in the Non-responders group (animal numbers 3, 7, 9 and 10) (Supplementary Fig. S4).

### LC-MS/MS analysis of Verteporfin in mice blood samples

Verteporfin present in plasma samples of mice at necropsy was analyzed at the Proteomics facility of Cornell University (http://www.biotech.cornell.edu/brc/proteomics-and-mass-spectrometry). The LC-MS method was developed for detecting Verteporfin with high selectivity and sensitivity. We used targeted IDA method for identification of the analyte. There were two forms of Verteporfin (depending on the position of CH_3_ and H) found in standard as well as in the samples having the retention time 9.86 and 10.2 min respectively. Verteporfin was analyzed in control, Responder and Non-responder samples (n = 3 each).

## Results

### Transcriptome analysis of control and Verteporfin treated EMCA cells

We treated Type 1 EMCA cells (HEC-1-A and HEC-1-B) with VP at 10 nM for 3 h. This short time frame (half-life of VP is approximately 5–6 hours) and the lower concentration of VP was chosen to allow us to examine immediate responses to VP and to minimize confounding effects associated with severe damage and cell death. Under these conditions, VP causes several changes in gene expression within 3 h of treatment. We used RNA-seq to measure the transcriptomes altered by VP treatment. We generated 220–270 million reads per lane, filtered reads to have high quality scores and mapped 75–80% of those reads to the human genome. In order to get an overview over similarities and dissimilarities between samples, a hierarchical clustering to the heatmap function based on the sample distances was used (Figs 1 and S1). The strict counts of genes after VP-treatment tended to be less than in their non-treated counterparts in both HEC-1-A and HEC-1-B (Supplementary Fig. S2A). The differential expression of genes between the two groups was compared using a negative binomial test (Fig. 2). When considering the effect of VP treatment in both cell lines, 841 genes were upregulated and 251 genes down regulated after VP treatment (Fig. 1B). Treatment with VP was found to modulate many different genes and the top 20 upregulated (Table 1) and top 20 downregulated genes (Table 2) in EMCA cells after VP treatment were tabulated. The GO annotations of the differentially expressed genes for the combined cell line data were provided (Table 3). Positive regulation of TGF-β1 production, regulation of lipoprotein metabolic process, cell adhesion, endodermal cell differentiation, formation and development, and integrin mediated signaling pathway were among the significantly associated terms.

**Table 1:** Top 20 upregulated genes in HEC-1-A and HEC-1-B cells after VP treatment, ranked by logFC.

Gene	Description	log_2_ Fold Change	lfcSE	padj
CYR61	Cysteine rich angiogenic inducer 61	3.170006	0.481229	1.858732e-08
VCAN	Versican	2.696764	0.386285	2.578137e-09
COL12A1	Collagen type XII alpha 1 chain	2.558625	0.52389	5.453066e-05
THBS1	Thrombospondin 1	2.451070	0.544776	1.680463e-04
ITGAV	Integrin subunit alpha V	2.361123	0.38602	3.119408e-07
TFPI2	Tissue factor pathway inhibitor 2	2.320415	0.43918	1.646115e-05
APP	Amyloid beta precursor protein	2.227067	0.366527	3.757190e-07
ANKRD1	Ankyrin repeat domain 1	2.224969	0.530091	1.007740e-03
GPC4	Glypican 4	2.211857	0.437625	4.312393e-05
SEMA3C	Semaphorin 3C	2.209851	0.417216	1.579824e-05
GANAB	Glucosidase II alpha subunit	2.200511	0.312619	1.903285e-09
EGR1	Early growth response 1	2.176855	0.41974	2.630788e-05
ATP6AP2	ATPase H+ transporting accessory protein 2	2.176744	0.321361	9.655713e-09
ITGB4	Integrin subunit beta 4	2.164238	0.35064	2.381589e-07
ADAM9	ADAM metallopeptidase domain 9	2.124249	0.380008	4.332059e-06
GBA	Glucosyl ceramidase beta	2.097261	0.283565	3.036162e-10
NUP210	Nucleoporin 210	2.096090	0.281061	2.108597e-10
DSG2	Desmoglein 2	2.088867	0.347257	5.337427e-07
OS9	OS9, endoplasmic reticulum lectin	2.085436	0.306665	8.375584e-09
COL4A2	Collagen type IV alpha 2 chain	2.066695	0.53173	2.780290e-03

Standard error of the logFC and p-value adjusted for false discovery rate are also included.

**Table 2:** Top 20 downregulated genes in HEC-1-A and HEC-1-B cells after VP treatment.

Gene	Description	log_2_ Fold Change	lfcSE	padj
KLKP1	Kallikrein pseudogene 1	−2.149389	−2.149771	0.000370
MROH2A	Maestro heat like repeat family member 2A	−2.038033	−2.110868	0.003762
PDE1B	Phosphodiesterase 1B	−1.913882	−2.089721	0.009297
SLC5A10	Solute carrier family 5 member 10	−1.907402	−2.038577	0.012501
HSPA6	Heat shock protein family A (Hsp70) member 6	−1.839579	−2.031518	0.011117
PPP1R27	Protein phosphatase 1 regulatory subunit 27	−1.799172	−1.928942	0.006376
DNLZ	DNL-type zinc finger	−1.796807	−1.914579	0.004334
MMP25	Matrix metallopeptidase 25	−1.766102	−1.909672	0.000267
CEND1	Cell cycle exit and neuronal differentiation 1	−1.759645	−1.908139	0.013744
EFCAB12	EF-hand calcium binding domain 12	−1.754972	−1.898853	0.008427
BRICD5	BRICHOS domain containing 5	−1.718193	−1.851035	0.000335
IL9RP3	Interleukin 9 receptor pseudogene 3	−1.717188	−1.840025	0.005862
AOC3	Amine oxidase, copper containing 3	−1.713105	−1.806662	0.006405
FTCD	Formimidoyltransferase cyclodeaminase	−1.711106	−1.799549	0.001850
MSLNL	Mesothelin-like	−1.704571	−1.797131	0.020615
RN7SL472P	RNA, 7SL, cytoplasmic 472, pseudogene	−1.703349	−1.795745	0.028688
MIR4521	MicroRNA 4521	−1.699961	−1.793331	0.005985
RBFOX3	RNA binding protein, fox-1 homolog 3	−1.678967	−1.77783	0.016016
SRPK3	SRSF protein kinase 3	−1.667756	−1.777185	0.007663
C9orf131	Chromosome 9 open reading frame 131	−1.667409	−1.766292	0.024349

**Table 3:** Gene Ontology for top 20 upregulated genes in HEC-1A and HEC-1B after VP treatment (GO term results with FDR-adjusted p < 0.05), sorted by fold enrichment. The p-value was adjusted for False Discovery Rate (FDR).

GO biological process complete	*H*. *sapiens* - REFLIST (21042)	upregulated (20)	upregulated (expected)	upregulated (Fold enrichment)	upregulated (over/under)	upregulated (raw P-value)	upregulated (FDR)
Positive regulation of transforming growth factor beta1 production	6	2	0.01	>100	+	2.39E-05	9.35E-03
Positive regulation of transforming growth factor beta production	17	2	0.02	>100	+	1.45E-04	3.07E-02
Regulation of multicellular organismal process	2827	12	2.69	4.47	+	1.55E-06	2.01E-03
Positive regulation of multicellular organismal process	1568	9	1.49	6.04	+	5.69E-06	3.87E-03
Regulation of transforming growth factor beta1 production	10	2	0.01	>100	+	5.62E-05	1.63E-02
Positive regulation of osteoblast proliferation	11	2	0.01	>100	+	6.64E-05	1.85E-02
Regulation of osteoblast proliferation	23	2	0.02	91.49	+	2.54E-04	4.56E-02
Regulation of lipoprotein metabolic process	13	2	0.01	>100	+	8.93E-05	2.37E-02
Regulation of protein metabolic process	2809	10	2.67	3.75	+	9.29E-05	2.38E-02
Cell adhesion mediated by integrin	24	3	0.02	>100	+	2.11E-06	2.36E-03
Cell adhesion	889	9	0.84	10.65	+	4.92E-08	2.56E-04
Biological adhesion	895	9	0.85	10.58	+	5.21E-08	2.04E-04
Positive regulation of macrophage activation	23	2	0.02	91.49	+	2.54E-04	4.51E-02
Endodermal cell differentiation	41	3	0.04	76.98	+	9.46E-06	5.10E-03
Cell differentiation	3636	12	3.46	3.47	+	2.27E-05	9.09E-03
Cellular developmental process	3728	12	3.54	3.39	+	2.94E-05	1.05E-02
Developmental process	5654	16	5.37	2.98	+	1.13E-06	1.61E-03
Endoderm formation	51	3	0.05	61.89	+	1.76E-05	7.25E-03
Endoderm development	76	3	0.07	41.53	+	5.53E-05	1.63E-02
Tissue development	1704	11	1.62	6.79	+	8.51E-08	2.22E-04
Anatomical structure development	5299	16	5.04	3.18	+	4.38E-07	8.55E-04
Formation of primary germ layer	116	4	0.11	36.28	+	4.52E-06	3.72E-03
Anatomical structure formation involved in morphogenesis	870	8	0.83	9.67	+	7.12E-07	1.11E-03
Anatomical structure morphogenesis	2104	12	2.00	6.00	+	5.98E-08	1.87E-04
Gastrulation	161	4	0.15	26.14	+	1.60E-05	7.13E-03
Embryonic morphogenesis	563	5	0.54	9.34	+	1.55E-04	3.11E-02
Embryo development	919	7	0.87	8.01	+	1.45E-05	6.89E-03
Multicellular organism development	4918	15	4.67	3.21	+	1.56E-06	1.87E-03
Multicellular organismal process	6807	16	6.47	2.47	+	1.66E-05	7.19E-03
Integrin-mediated signaling pathway	92	3	0.09	34.31	+	9.59E-05	2.34E-02
Cellular response to stimulus	6741	15	6.41	2.34	+	1.02E-04	2.41E-02
Extracellular matrix organization	323	8	0.31	26.06	+	3.66E-10	5.72E-06
Extracellular structure organization	366	8	0.35	23.00	+	9.59E-10	7.50E-06
Cell-matrix adhesion	123	3	0.12	25.66	+	2.21E-04	4.12E-02
Positive regulation of cellular amide metabolic process	133	3	0.13	23.73	+	2.77E-04	4.76E-02
Response to tumor necrosis factor	218	4	0.21	19.30	+	5.10E-05	1.53E-02
Response to chemical	4323	12	4.11	2.92	+	1.35E-04	3.02E-02
Striated muscle tissue development	289	4	0.27	14.56	+	1.49E-04	3.07E-02
Muscle tissue development	302	4	0.29	13.94	+	1.76E-04	3.44E-02

**Figure 3:**
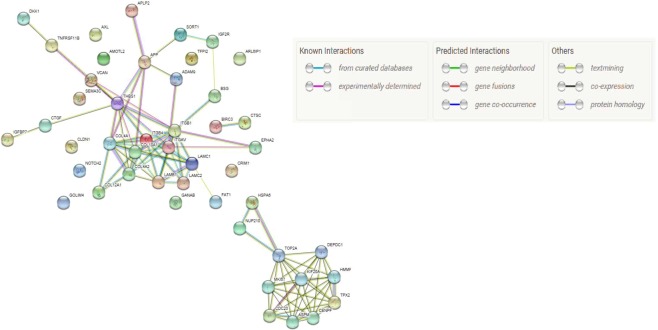
STRING network of protein-protein interactions between top 50 down-regulated and up-regulated genes in HEC1-B by adjusted p-value.

When comparing DESeq-derived differentially-expressed genes by cell line, HEC-1-B showed the most marked change in RNA expression after VP treatment (Supplementary Fig. S2B, S2C). While many genes with low mean normalized count showed a high logFC after VP treatment, more genes with high mean normalized count showed general downregulation in HEC-1-B (Fig. 3 and Supplementary Tables S1, S2). For the analysis using only HEC-1-B samples, 8582 genes had differential expression with P < 0.05 after false discovery rate (FDR) correction. Of these, 3235 were downregulated and 5271 were upregulated in VP treated cells as compared to control cells. In HEC-1-B, differentially expressed genes with lowest p-values were VCAN, COL4A1, LAMC1, HSPA6, COL4A2, COL12A1, ITGAV, SPON1, KIF20A, ASPM. Notable was the fact that versican (VCAN), a gene that codes for a large extracellular matrix proteoglycan, had the lowest adjusted p-value (2.45e-216) and large absolute log2FC (log2FC = −3.463, lfcSE = 0.109) amongst all differentially expressed genes in HEC-1-B after VP-treatment. The tumor microenvironment has been found to contribute to VCAN mRNA expression (mostly in cleaved forms) in multiple tumor cell lines^26,27^.

### Gene co-expression

The VP-treated HEC-1-A cell line is more similar to VP-treated HEC-1-B in terms of transcriptomic gene expression; in fact, they are closer to each other than the control HEC-1-A and the control HEC-1-B (Figs 1 and 2). Functional enrichment analysis of differentially expressed genes in Type 1 cells after VP revealed that the extracellular matrix (ECM) organization Gene Ontology was the most significant (FDR p-value = 2.88e-15) (Table 3).

### Protein product co-expression network

The resulting protein products of differentially expressed transcripts after VP-treatment were investigated for possible interaction with each other using STRING, which uses prior biological data (Figs 3 and S3, Supplementary Table S3)^28^. As the HEC-1-B cell line exhibited the most differential expression after VP-treatment, it was posited that the most differentially expressed genes would have strong interactions with each other. CDC23 and BUB1B, two genes crucially involved in mitotic checkpoint progression, were found to be the pair that had the best association from STRING among genes that were significantly differentially expressed in HEC-1-B after VP treatment (Supplementary Table S3). We obtained the Pfam protein domains and KEGG (Kyoto Encyclopedia of Genes and Genomes) pathways mostly associated with the differentially expressed genes using STRING (Fig. 3, Supplementary Table S5). Significantly enriched KEGG^29^ pathway protein products (Top 25) from genes differentially expressed in HEC-1-B after VP treatment were tabulated in Supplementary Table S4. Out of 124 genes in the KEGG cell cycle pathway (hsa-4110), 92 of them exhibited altered expression (adjusted p-value < 0.05) in HEC-1-B after treatment in VP; the top 25 are shown in Table 4. Enrichment in cell cycle genes was not present to the same degree in HEC-1-A. Further, we validated the RNASEQ data using qRTPCR in HEC-1-B cells treated with VP and observed that HSPA6 and C2orf88 genes were upregulated whereas CYR61, THBS1and ANKRD1 were downregulated after VP treatment. Based on the protein product co-expression data, we checked the expression of BUB1B, CDC23, MAD2L1, BUB3 and CDC27 genes and observed that these were downregulated after VP treatment in HEC-1-B cells. (Supplementary Table S6.) Similarly, we checked the expression of cell cycle genes CCRK, CDK2, CCND1, CCNE1 and E2F1 in both VP-treated EMCA cells (Supplementary Table S7) and VP-treated mice tumors (Supplementary Table S8) and observed that VP effectively inhibited the cell cycle genes. The downregulation of cell cycle proteins was established in VP-treated EMCA cells (Supplementary Fig. S5A). Further, we tested the role of YAP in regulating cell cycle proteins. YAP-knockdown in HEC-1-B cells by shYAP treatment resulted in downregulation of cell cycle proteins CCRK, CDK2 and Cyclin D1 (Supplementary Fig. S5B). These results show that VP is effective in inhibiting cell cycle both at transcription and translation levels. Interestingly, we also observed that VP downregulates pluripotency marker Oct4 either *in vitro* and *in vivo* (Supplementary Fig. S5C and S5D).

**Table 4:** Genes altered with an FDR adjusted p-value < 0.05 that belong to KEGG cell cycle pathways in HEC-1-B.

ID	Gene	Biotype	Chromosome	Description	Base Mean	log2Fold Change	lfcSE	padj
ENSG00000164611	PTTG1	protein coding	5	Pituitary tumor-transforming 1	1266.799095	−2.891629	0.153204	8.33E-77
ENSG00000157456	CCNB2	protein coding	15	Cyclin B2	712.149481	−3.302557	0.174789	1.08E-76
ENSG00000169679	BUB1	protein coding	2	BUB1 mitotic checkpoint serine/threonine kinase	749.048122	−2.559155	0.166587	5.09E-51
ENSG00000145386	CCNA2	protein coding	4	Cyclin A2	519.014978	−2.649113	0.177117	2.50E-48
ENSG00000170312	CDK1	protein coding	10	Cyclin dependent kinase 1	1000.755561	−2.648046	0.1822	9.28E-46
ENSG00000166851	PLK1	protein coding	16	Polo like kinase 1	597.264747	−2.390371	0.16569	4.52E-45
ENSG00000156970	BUB1B	protein coding	15	BUB1 mitotic checkpoint serine/threonine kinase B	766.341487	−2.396273	0.177853	2.06E-39
ENSG00000100297	MCM5	protein coding	22	Minichromosome maintenance complex component 5	1912.808686	−1.775605	0.145411	1.55E-32
ENSG00000076003	MCM6	protein coding	2	Minichromosome maintenance complex component 6	2258.121144	−1.561124	0.128417	2.93E-32
ENSG00000166483	WEE1	protein coding	11	WEE1 G2 checkpoint kinase	1064.819728	−1.837664	0.154052	4.46E-31
ENSG00000104738	MCM4	protein coding	8	Minichromosome maintenance complex component 4	3564.827682	−1.513408	0.128823	3.52E-30
ENSG00000164754	RAD21	protein coding	8	RAD21 cohesion complex component	5391.58818	−1.250919	0.106903	6.02E-30
ENSG00000080839	RBL1	protein coding	20	RB transcriptional corepressor like 1	678.135332	−1.958297	0.167687	8.47E-30
ENSG00000164109	MAD2L1	protein coding	4	Mitotic arrest deficient 2 like 1	409.014019	−2.418097	0.209223	3.83E-29
ENSG00000094804	CDC6	protein coding	17	Cell division cycle 6	1542.735216	−1.598088	0.143578	3.62E-27
ENSG00000149554	CHEK1	protein coding	11	Checkpoint kinase 1	745.52042	−1.611426	0.151025	5.26E-25
ENSG00000158402	CDC25C	protein coding	5	Cell division cycle 25C	161.535519	−3.008824	0.2819	1.45E-24
ENSG00000073111	MCM2	protein coding	3	Minichromosome maintenance complex component 2	4500.5434	−1.518689	0.146626	1.27E-23
ENSG00000105810	CDK6	protein coding	7	Cyclin dependent kinase 6	568.901075	−1.704795	0.165148	1.90E-23
ENSG00000198176	TFDP1	protein coding	13	Transcription factor Dp-1	2356.504771	−1.25606	0.129064	6.35E-21

### Effect of Verteporfin *in vivo* in nude mice

**Figure 4:**
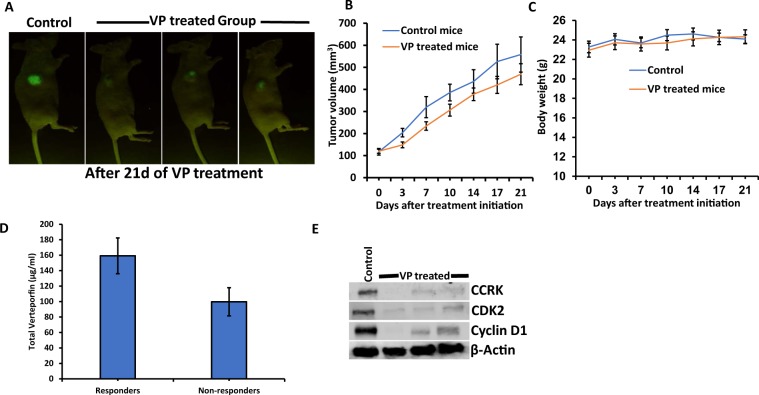
(**A**) NCr nude mice showing tumor regression after VP treatments in a subcutaneous model after 21 days. n = 10/group. (**B**) Tumor volume growth curve and (**C**) Body weight curves in control and VP treated mice. (**D**) Quantitation of VP in mice plasma samples by LC-MS/MS analysis. (**E**) Western blot showing VP-induced inhibition of cell cycle proteins in subcutaneous tumors of mice. Error bars indicate Mean ± SEM.

Since we observed significant cytotoxicity and induction of apoptosis by VP in EMCA cells, we employed a preclinical xenograft mouse model of EMCA to investigate the *in vivo* efficacy of VP on HEC-1-B cells in NCr nude mice. HEC-1-B GFP cells were injected into mice by subcutaneous (SC) administration. IP administration in this model achieved tumor regression (Fig. 4A,B) and importantly did not hasten time to euthanasia or decrease in weight between control and treated animals (Fig. 4C). These results show that VP is not toxic to mice. Based on the tumor volume data, we classified VP-treated mice into Responders and Non-responders (See Materials and Methods). Mean tumor volume in control mice was 558.34 mm^3^. Responders recorded mean tumor volume of 369.13 mm^3^ (33.89% decrease compared to control) whereas Non-responders had tumor volume of 619.15 mm^3^ (10.89% increase compared to control) (Supplementary Fig. S4A, S4C). Similarly, mean tumor weight of control mice was 0.302 g. In case of Responders, mean tumor weight decreased by 45.36% (0.165 g) compared to an increase of 18.54% (0.358 g) in Non-responders (Supplementary Fig. S4B). In order to check the concentration of VP in mouse blood, we analyzed their plasma samples using the LC-MS/MS method. Verteporfin was not detected in control samples and it was detected in Responder and Non-responder samples with good signal intensity. Responders had high amounts of VP (159.25 µg/ml) compared to non-responders (99.75 µg/ml) (Fig. 4D). These results indicate the bio-availability of VP and its effect on tumor regression. For further analyses, we used tumor samples of responders. Western analysis of control and VP treated tissues shows that VP is probably inducing tumor regression by acting upon cell cycle proteins (Fig. 4E). In accordance with our *in vitro* results, we observed a moderate effect of VP on EMCA tumor growth.

## Discussion

Verteporfin is a porphyrinic photosensitizer clinically used for the photodynamic treatment of age-related macular degeneration. YAP is a transcriptional co-activator and a potent oncogene^30–32^. Inhibition of the Hippo pathway leads to YAP activation, nuclear localization and cell proliferation in most cell types. The interaction of YAP with the TEAD family of transcriptional activators leads to DNA binding and transcription. YAP is amplified in many human cancers including breast, esophageal, hepatocellular, malignant mesothelioma, medulloblastoma and ovarian^30,33–36^. Based on the recent reports, VP is found to be a multi-target drug interacting with several proteins implicated in major cellular processes^37^. However, VP has been shown to inhibit autophagy in the absence of light activation both *in vitro* and *in vivo*^38–40^. Given the highly reactive nature of VP with light, Konstantinou *et al*.^41^ investigated the proposed mechanisms of non-light activated VP effects and suggested that VP-induced high molecular weight protein complexes (HMWC) require the presence of light. They observed that both singlet oxygen and radical generation mediate the formation of cross-linked oligomers and HMWC by VP in the presence of light. However, non-light activated, and light-independent VP effects have been demonstrated by several authors^42–44^. Verteporfin was identified as a potent inhibitor of cell growth in retinoblastoma cells, disrupting YAP-TEAD signaling and pluripotential marker Oct4^42^. Verteporfin can also reverse the paclitaxel resistance induced by YAP over-expression in HCT-8/T cells without photoactivation through inhibiting YAP expression^43^. Cytotoxic effects of VP were mediated by light-independent production of reactive oxygen species and its anti-leukemic effects were also exerted *in vivo* without photoirradiation^44^. Similarly, Donohue *et al*. proposed that exposure to bright light could be harmful for VP-treated animals^39^. Based on these reports, in order to study the non-light activated effects of VP, we treated EMCA cells with VP for 3 h in the dark at 37 °C, followed by lysis with overhead fluorescence ambient laboratory lighting. Similar to previous findings, we observed maximum effect of VP in EMCA cells in inhibiting cell cycle and inducing apoptosis^17^. Based on the present results of RNA-seq analysis, VP induced modification of several genes belonging to different pathways, altering the transcriptomic etiology of EMCA cells.

Currently, VP has been widely used to understand the role of YAP1 in various cancers and is used for the treatment of various cancers^17,45^. Very few studies focused on the RNASeq analysis of EMCA pathophysiology. Mi *et al*. identified that the RACGAP1-STAT3-survivin signaling pathway is required for the invasive phenotype of uterine carcinosarcoma by applying RNA-Seq analysis to prospectively collected uterine carcinosarcoma tumor samples from patients^46^. Chen *et al*. studied the lncRNA transcriptome of endometrial cancers and adjacent normal endometrium from the same patients and compared with transcriptomes of other gynecologic malignancies including ovarian and cervical cancers^47^. The RNA-seq data of uterine corpus endometrial carcinoma (UCEC) samples identified potential genes associated with the development of UCEC. This group downloaded UCEC RNA-seq data from The Cancer Genome Atlas database^48^ and identified multiple key genes in UCEC and clinically relevant small molecule agents, thereby improving the understanding of UCEC and expanding perspectives on targeted therapy for this type of cancer. So far, there have been no reports on transcriptome of EMCA cells under *in vitro* conditions. In our study, we used RNA-seq and bioinformatic methods to better understand the EMCA cellular response to VP at the transcriptomic level, and possibly gain insight into the molecular mechanisms that might account for the pathophysiology of EMCA. Moreover, the underlying transcriptional response to VP suggests that EMCA cells carry out their function through different gene regulatory mechanisms. The results of the present study indicated that multiple differentially expressed genes are associated with EMCA pathophysiology. Differential expression analysis revealed changes in the expression patterns of many protein-coding genes previously reported to be involved in other cancers (Tables 3–4). Genes modulated by VP treatment include the genes related to positive regulation TGFβ1, lipoprotein metabolic processes, cell adhesion mediated by integrins, endodermal cell differentiation and extracellular matrix organization.

Even though we performed RNA-Seq analysis for both HEC-1-A and HEC-1-B cell lines, combining data related to both cell lines led to a result with not many significant pathways or gene-gene interactions. As in the PCA plot (Supplementary Fig. S1), the transcriptomic distance between HEC-1-A and HEC-1-B is too great to come up with many combined pathways. Hence, we focused our further analysis on HEC-1-B cell line data. Previously, we have shown that VP inhibits cell cycle progression of Type 1 EMCA cells^17^, hence we analyzed KEGG cell cycle pathways in HEC-1-B. Out of 124 genes in the KEGG cell cycle pathway, 92 of them exhibited altered expression after VP treatment. These results also corroborate with our *in vivo* studies, as we observed that tumor regression in mice is induced by inhibition of cell cycle proteins. Amongst all genes that were differentially expressed in HEC-1-B after VP treatment, naïve of biological annotation, mRNA transcripts of VCAN were most likely to be downregulated with VP treatment (Supplementary Fig. S2). The role of Versican in cell adhesion, migration, and proliferation has been extensively studied^49,50^, and in cancer, stromal expression of VCAN was strongly correlated with disease recurrence^26^. The downregulating effect of VP treatment on VCAN expression for the HEC-1-B cell line is promising, but the precise mechanism of this effect remains to be elucidated, especially considering the fact that a contrary upregulating effect on VCAN expression was found in HEC-1-A, or when both HEC-1-A and HEC-1-B were analyzed together (Table 1). Cyclin-dependent kinase 20 (CDK20) or CCRK, is a member of CDK family with strong linkage to human cancers. Recent studies reported the consistent overexpression of CCRK in cancers arising from brain, colon, liver, lung and ovary. The signaling molecules perturbed by CCRK are divergent and cancer-specific, including the cell cycle regulators CDK2, cyclin D1, cyclin E and RB in glioblastoma, ovarian carcinoma and colorectal cancer and lung cancer^51^. Overexpression of CCRK increases cyclin D1 expression, which suggests the role of CCRK in the control or cell proliferation via regulation of cyclin D1 expression^52^. Recently Zapiecki *et al*. showed that significantly higher expression of cyclin D1 and cyclin E was detected in patients dying from endometrial cancer^53^. Cyclin D1 plays an important role in LSD1-regulated estrogen-induced endometrial cancer cell proliferation^54^. This group also verified the positive correlation between LSD1 and cyclin D1 in endometrial cancer tissues by immunohistochemistry. In conformity with previous findings, our studies also indicate that inhibition of cyclin D1, CCRK and CDK2 are involved in inhibition of growth and proliferation in EMCA cells and tumor regression in mice. Normal adult stem cells and cancer stem cells maintain expression of Oct3/4, consistent with the stem cell hypothesis of carcinogenesis. Hence, a strategy to target “cancer stem cells” is to suppress the Oct4 gene expression^55^. Recently Ding *et al*. studied the characteristics of CD133^+^ cells isolated from endometrial cancer^56^. They proposed that CD133^+^ cells increased expression of embryonic stem cells markers including Oct4, Nanog, Sox2 than CD133^−^ cells. Verteporfin was shown to downregulate Oct4 expression in retinoblastoma cells^41^. Our findings corroborate with previous results under both *in vitro* and *in vivo* conditions and also suggest a mechanism of action of VP.

The administration of VP in subcutaneous mouse model identified both Responders and Non-responders to this treatment. Our results show that VP is not toxic to mice without significant effect on their body weight. Visudyne^®^, the commercial liposomal formulation of VP was developed for photodynamic therapy. Based on the calculations of low verteporfin-to-lipid ratio of Visudyne, Donohue *et al*. administered 20 mg/kg VP and observed that VP tumor accumulation following i.v. administration was very poor in pancreatic ductal adenocarcinoma (PDAC) mouse model^39^. To increase VP tumor accumulation, they developed an alternate micellar formulation with DSPE-mPEG2000 dissolved in PBS. They observed that VP inhibited autophagy *in vivo* but did not reduce tumor volume or increase survival as a single agent in PDAC mouse model. They also observed that VP in combination with gemcitabine moderately reduced tumor growth and enhanced survival compared to gemcitabine alone. Similar to their findings Zhao *et al*. demonstrated that VP in combination with a pan-RAF inhibitor LY3009120 significantly enhanced tumor regression in KRAS-mutant pancreatic cancer^57^. Our results support their findings as we observed that VP failed to show antitumor activity in non-responders, even though the drug accumulated at the tumor site based on our LC-MS/MS analysis. These results warrant further standardization of solubility of VP, dosing schedule of VP, route of administration, as well as use of VP in combination with other chemotherapeutic agents for effective treatment in EMCA. On-going experiments in our laboratory include development of orthotopic model of EMCA, administration of VP as mPEG-PLGA nanoparticles and IV administration of VP with pharmacokinetic studies. The present study revealed multiple key genes of pathological significance in EMCA as well as mechanism of activity of VP with the ultimate goal of improving our understanding of molecular profile of EMCA which expands our perspective on targeted therapy for this cancer. Taken together our results suggest that VP may be repurposed for the treatment of advanced endometrial cancer.

## Supplementary information

Supplementary Data

## Data Availability

Additional information for reproducing the results described in the article is available upon reasonable request and subject to a data use agreement.
