# Supplementary material for: Differential gene expression induced by Verteporfin in endometrial cancer cells
Source: Sci Rep. 2019 Mar 7;9:3839. doi: 10.1038/s41598-019-40495-9 (PMC6405995; doi:10.1038/s41598-019-40495-9)
Supplement: Supplementary file 1 — Supplementary Data [file 41598_2019_40495_MOESM1_ESM.pdf]

Differential gene expression induced by Verteporfin in endometrial cancer cells

Gahyun Bang[1]#, Venkata Ramesh Dasari[2#], Dokyoon Kim[1, 3], and Radhika P Gogoi[2,4*]

[1]Biomedical and Translational Informatics Institute, Geisinger, Danville, PA, USA, [2]Weis Center for Research, Geisinger Clinic, Danville, PA, USA, [3]Huck Institute of the Life Sciences, Pennsylvania State University, University Park, PA, USA, [4]Geisinger Medical Center, Danville, PA, USA

# Equal contribution.

*Corresponding author:
Radhika Gogoi, MD, PhD
Abigail Clinical Investigator
Weis Center for Research
Geisinger Clinic
100 North Academy Avenue
Danville, PA 17822, USA
Email: rpgogoi@geisinger.edu
Phone: 570-214-9016
Fax: 570-214-3074

Supplementary Data

Supplementary Figure S1

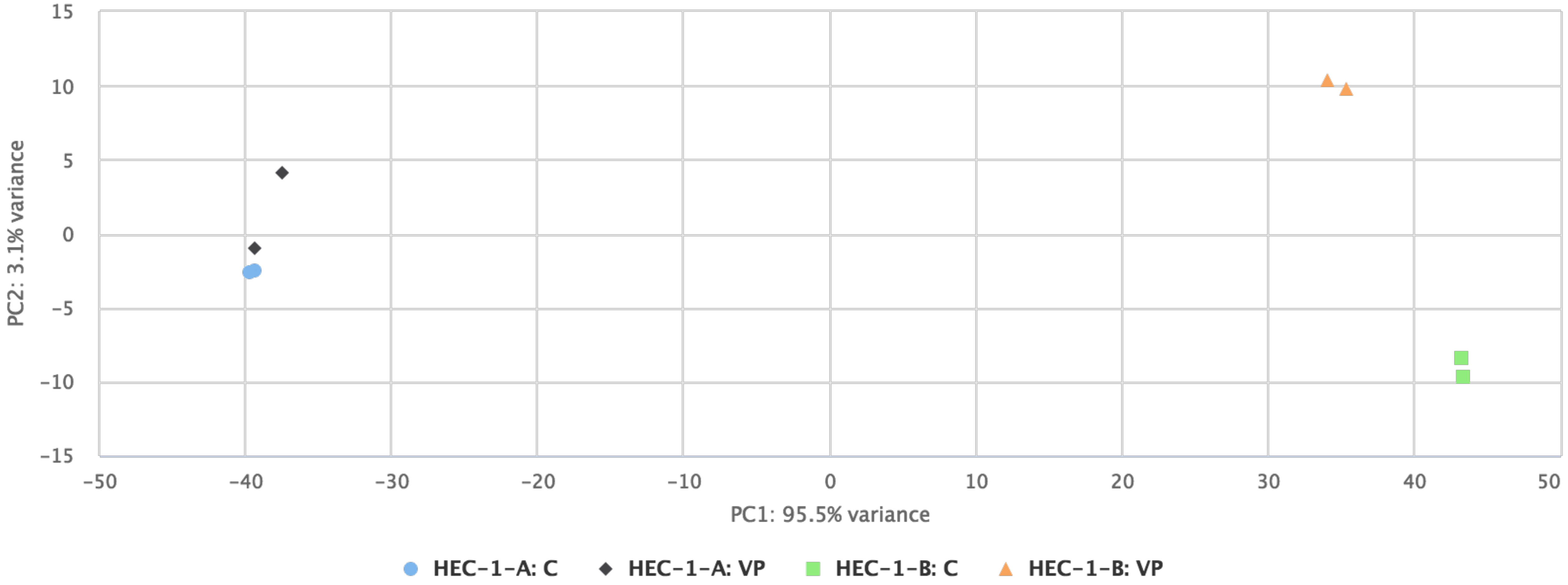

Supplementary Figure S2

A

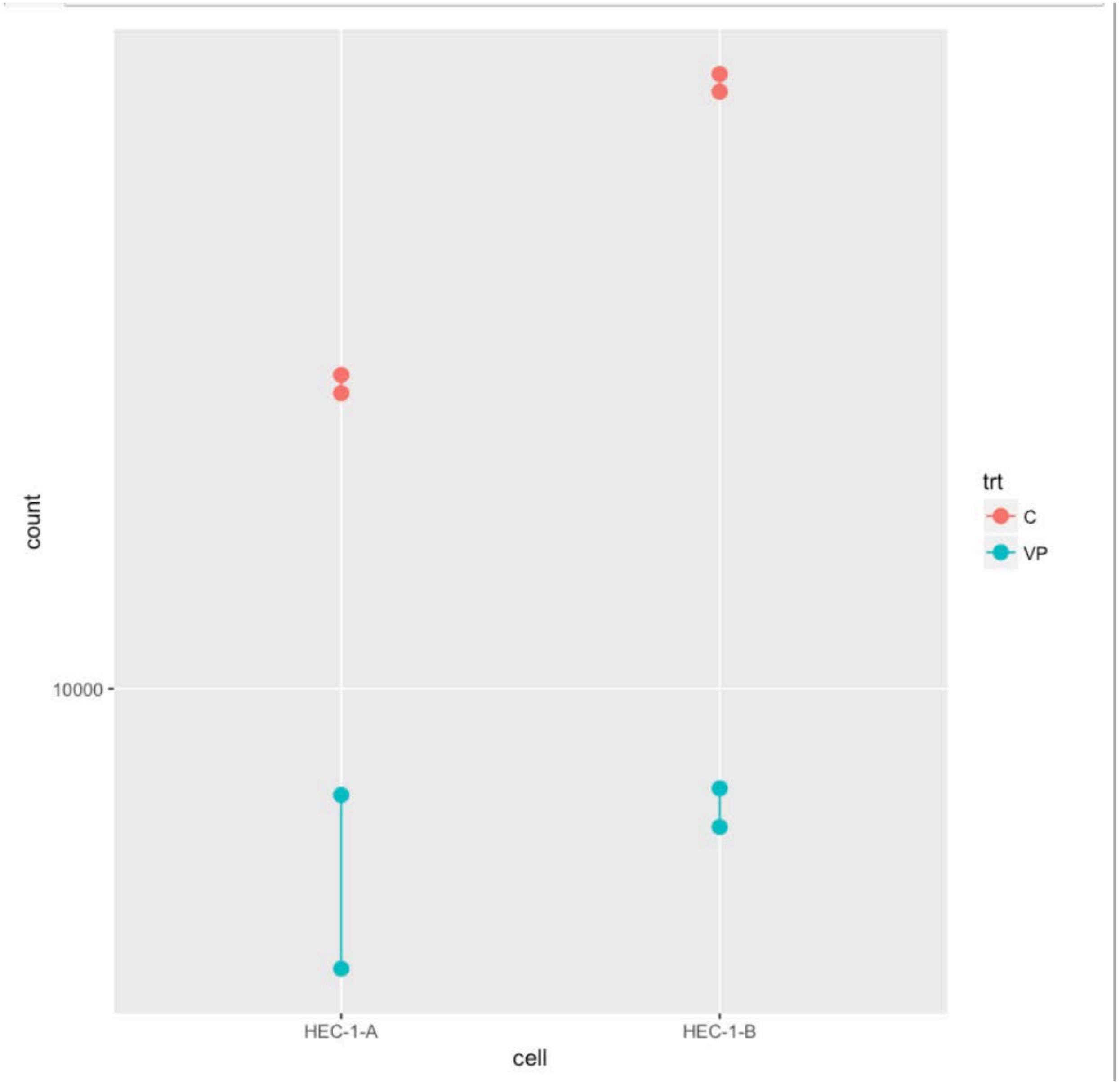

Supplementary Figure S2

B

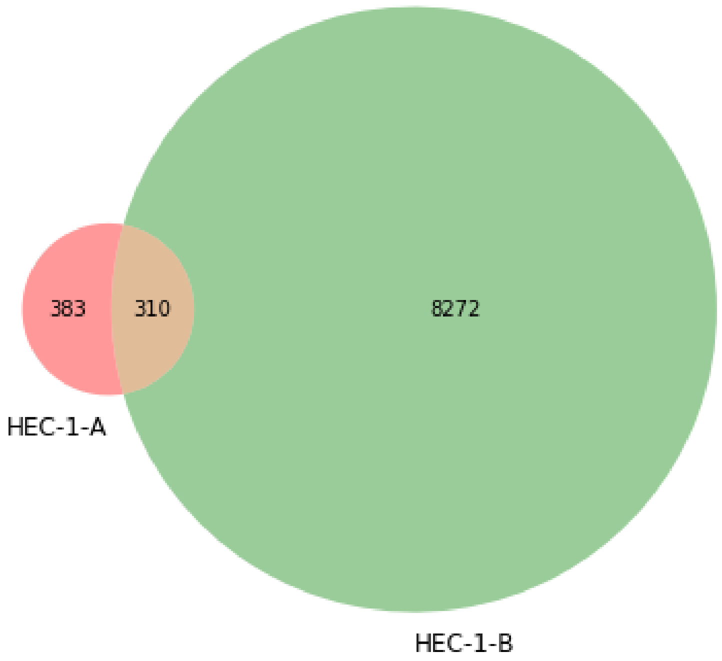

C

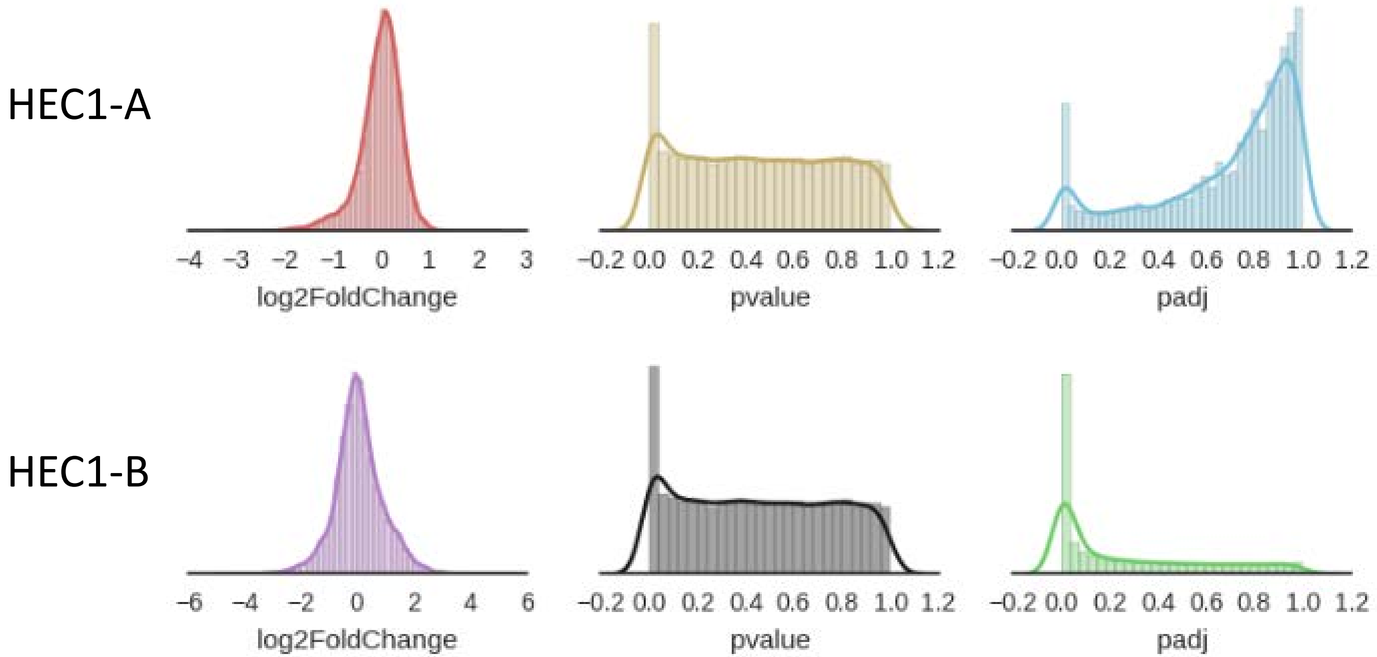

Supplementary Figure S3

A

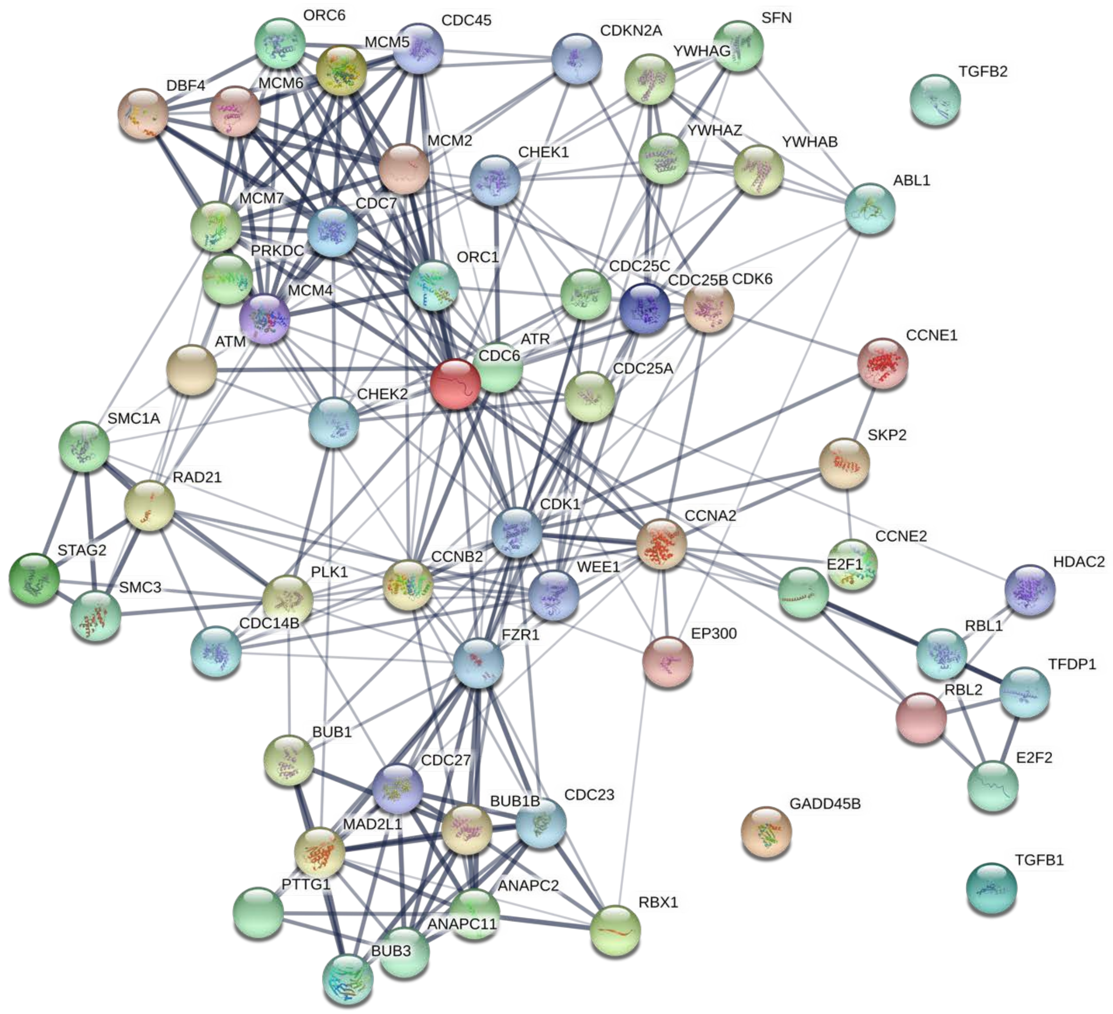

Supplementary Figure S3

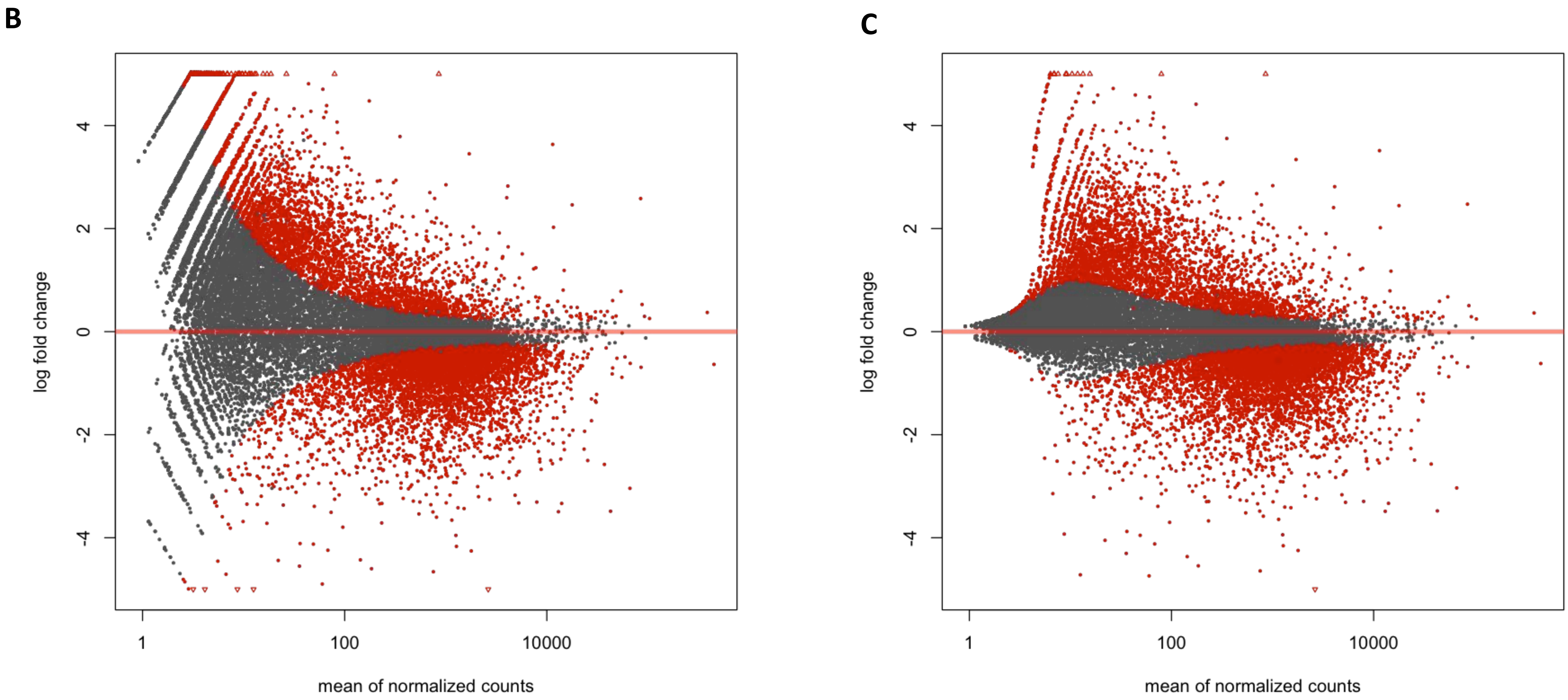

Supplementary Figure S4

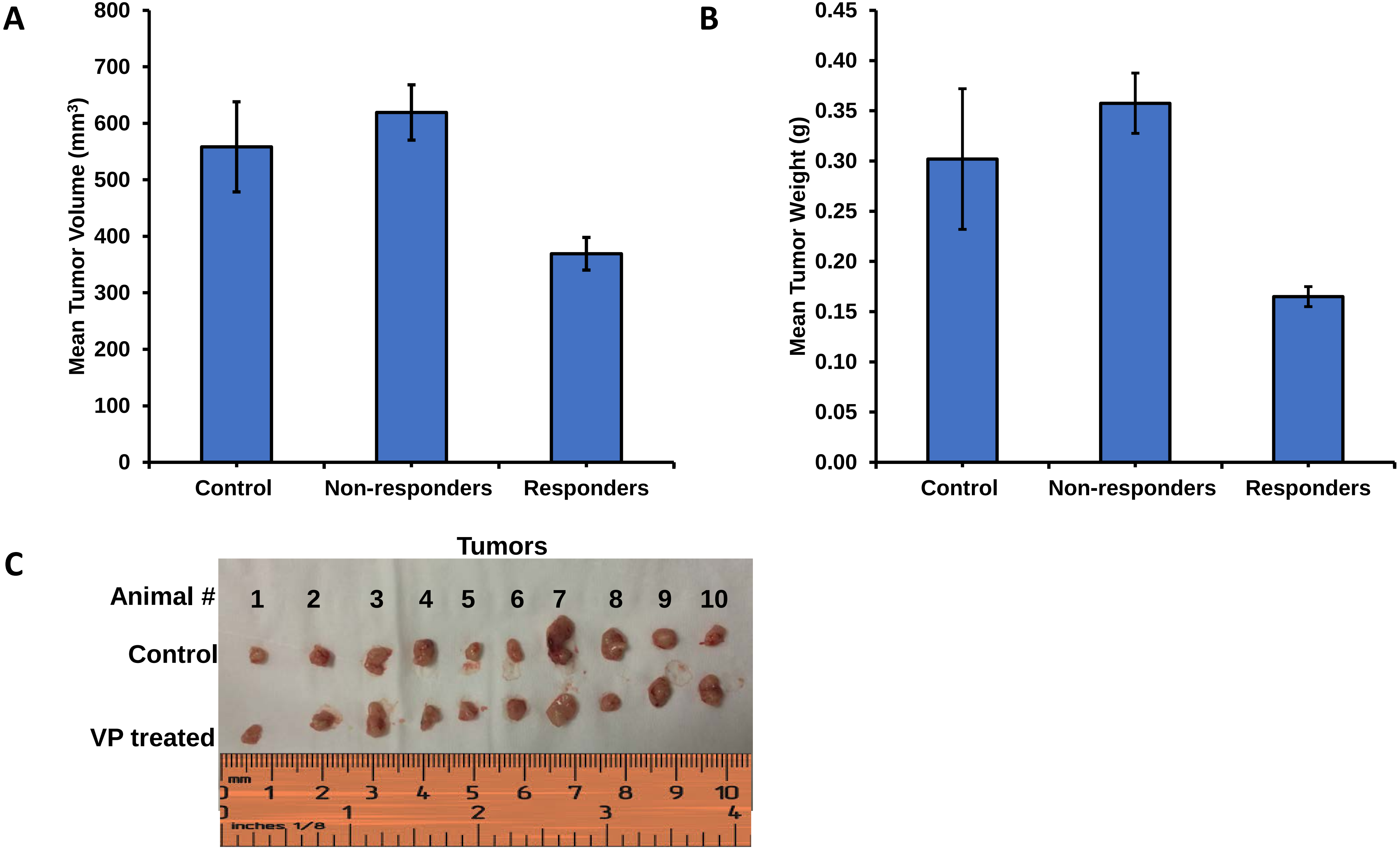

Supplementary Figure S5

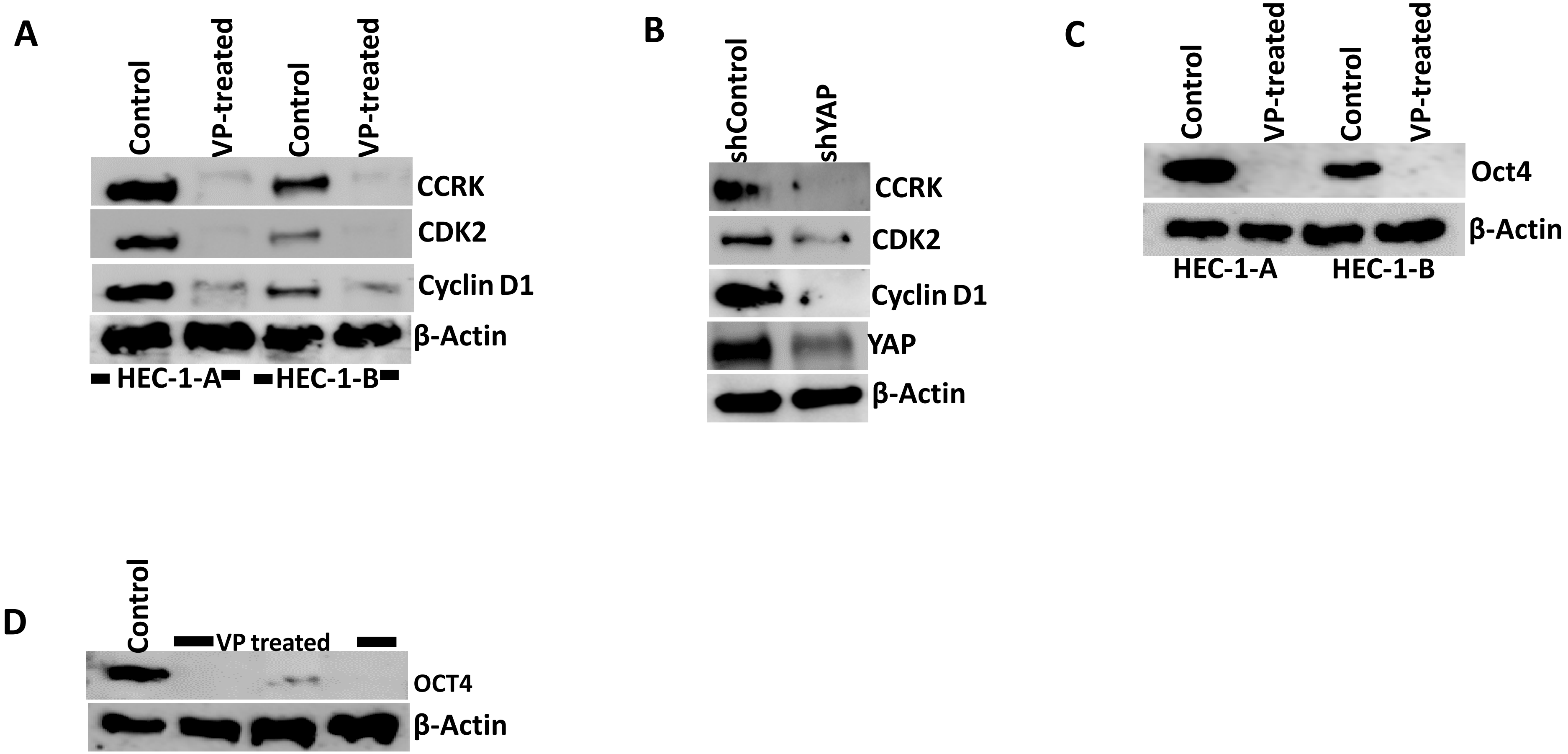

Supplementary Figure Legends

Supplementary Figure S1. PCA plot of HEC-1-A and HEC-1-B: PC1 explains 95.5% of the variance while PC2 explains 3.1% of the variance in a regularized log transform of normalized RNAseq data.

Supplementary Figure S2. (A) Count plot of normalized counts with lines connecting cell lines. As DESeq takes into account the cell line effect, the difference being tested is more closely depicted here. (B) Venn diagram showing total number of significantly differentially expressed genes in HEC-1-A and HEC-1-B cell lines. Differentially expressed genes in common with padj>0.05 shown here. (C) Quick overview of differentially expressed genes, fold changes and p-values (control vs. VP-treated).

Supplementary Figure. S3. (A). Experimentally-derived protein-protein interaction STRING network for the protein products of top differentially expressed genes for HEC-1B after VP treatment. MA-plots of distribution of the estimated coefficients in the model across all genes for HEC-1B cell line. The logFC after VP treatment was plotted on the y-axis and the average of the counts normalized by size factor plotted on the x-axis. **B**. plot of changes induced by treatment – unshrunk average of the counts normalized by size factor. Each gene is represented with a point; genes with a padj of less than 0.1 are in red. **C**. MA-plot using counts with shrink function used on log2FC for the comparison of VP and C samples.

Supplementary Figure S4. Bar graphs showing (A) Tumor volume and (B) tumor weight curves in both Responders and Non-responders in VP-treated mice. *(C). Tumors of both control and VP-treated mice.* Error bars indicate Mean ± SEM.

Supplementary Figure S5. (A). Western blot showing VP-induced inhibition of cell cycle proteins in EMCA cells. (B). Western blot analysis showing the effect of YAP knockdown on cell cycle proteins in HEC-1-B cells. Western blot analysis showing the effect of VP on Oct4 in (C) EMCA cells and (D) mouse subcutaneous tumors.

Supplementary Table S1. Top 20 upregulated genes in HEC-1-B cells after VP treatment. Standard error of the logFC and p-value adjusted for false discovery rate are also included:

Gene	Description	log2FoldChange	lfcSE	padj
HSPA6	Heat shock protein family A (Hsp70) member 6	5.356512	0.218491	2.08E-129
NECAB2	N-terminal EF-hand calcium binding protein 2	4.480058	0.458063	4.06E-21
C2orf88	Chromosome 2 open reading frame 88	4.15286	0.345405	1.41E-31
AC063976.7	*	3.978924	0.468799	4.42E-16
FAM124A	Family with sequence similarity 124 member A	3.825024	0.51893	2.58E-12
RP11-379F4.4	*	3.766931	0.45674	3.16E-15
RP11-182N22.9	*	3.719122	0.439024	5.04E-16
MROH2A	Maestro heat like repeat family member 2A	3.654299	0.401217	2.10E-18
KRT15	Keratin 15	3.63973	0.260933	2.88E-42
CYP4F3	Cytochrome P450 family 4 subfamily F member 3	3.565897	0.45891	1.34E-13
PRSS37	Protease, serine 37	3.561572	0.585642	1.20E-08
PDE1B	Phosphodiesterase 1B	3.54203	0.492056	8.84E-12
SERPINE3	Serpin family E member 3	3.49608	0.391443	1.01E-17
AC114812.9	*	3.325213	0.548638	1.36E-08
ADAM20	ADAM metallopeptidase domain 20	3.319796	0.52968	3.92E-09
RP11-385D13.1	*	3.280768	0.661252	4.92E-06
CTD-2083E4.5	*	3.247972	0.380907	3.19E-16
KIRREL2	Kin of IRRE like 2 (Drosophila)	3.221068	0.644197	4.08E-06
NEAT1	Nuclear paraspeckle assembly transcript 1 (non...	3.210791	0.435211	2.46E-12
C1QTNF7	C1q and tumor necrosis factor related protein 7	3.208637	0.432881	1.91E-12

*New Gene

Supplementary Table S2. Top 20 downregulated genes in HEC-1-B cells after VP treatment.

Standard error of the logFC and p-value adjusted for false discovery rate are also included:

Gene	Description	log2FoldChange	lfcSE	padj
CYR61	Cysteine rich angiogenic inducer 61	-4.880134	0.303638	6.35E-56
THBS1	Thrombospondin 1	-4.56434	0.192111	1.54E-121
ANKRD1	Ankyrin repeat domain 1	-4.301334	0.329177	3.65E-37
COL12A1	Collagen type XII alpha 1 chain	-4.210756	0.140174	1.56E-194
DHRS2	Dehydrogenase/reductase 2	-4.114623	0.341799	1.19E-31
PRSS35	Protease, serine 35	-4.112676	0.47337	8.20E-17
KIF20A	Kinesin family member 20A	-4.107755	0.162174	4.52E-138
HMMR	Hyaluronan mediated motility receptor	-3.900449	0.158396	1.59E-130
CTD-2510F5.4	Nan	-3.73182	0.428318	6.64E-17
DKK1	Dickkopf WNT signaling pathway inhibitor 1	-3.605873	0.161435	1.70E-107
SOX2	SRY-box 2	-3.567507	0.532713	2.60E-10
CTSV	Cathepsin V	-3.564999	0.264575	1.78E-39
DLGAP5	DLG associated protein 5	-3.532073	0.186416	1.59E-77
EDN1	Endothelin 1	-3.482653	0.476836	4.19E-12
COL4A1	Collagen type IV alpha 1 chain	-3.471255	0.111152	4.35E-210
PRR11	Proline rich 11	-3.46882	0.18829	2.57E-73
VCAN	Versican	-3.463459	0.109227	2.45E-216
PLCL1	Phospholipase C like 1	-3.45863	0.378973	1.79E-18
DEPDC1	DEP domain containing 1	-3.444251	0.169346	3.39E-89
IGFBP7	Insulin like growth factor binding protein 7	-3.380419	0.168105	3.28E-87

Supplementary Table S3. Protein product co-expression table for genes differentially expressed in HEC-1B after VP treatment (experimentally determined links from the literature) from STRING:

#Gene1	#Gene2	Experimentally determined interaction
CDC23	BUB1B	0.997
MAD2L1	BUB1B	0.997
CDC23	ANAPC2	0.997
CDC27	CDC23	0.997
CDC27	ANAPC2	0.997
MCM2	MCM6	0.992
ANAPC2	BUB1B	0.991
BUB3	BUB1B	0.989
MCM6	MCM5	0.989
BUB1	BUB1B	0.989
ANAPC11	ANAPC2	0.989
MCM2	MCM4	0.989
BUB3	BUB1	0.989
ORC1	CDC6	0.989
CDC27	BUB1B	0.989
MCM4	MCM5	0.989
MCM7	MCM2	0.989
MCM7	MCM6	0.989
MCM2	CDC7	0.989
MCM7	MCM5	0.989
MCM6	MCM4	0.989
MCM7	MCM4	0.989
MCM2	MCM5	0.989
CDC45	MCM6	0.988
SMC3	RAD21	0.988
ORC1	MCM6	0.988
CDC45	MCM7	0.988
ORC1	MCM2	0.988
FZR1	CDC23	0.988
ORC1	MCM4	0.984
TFDP1	E2F1	0.983
SMC1A	RAD21	0.980
ORC1	MCM5	0.980

FZR1	ANAPC2	0.976
BUB1	MAD2L1	0.970
CDC45	MCM4	0.969
MCM4	CDC7	0.969
BUB3	MAD2L1	0.969
CDC45	MCM2	0.968
CDC23	MAD2L1	0.967
SMC3	SMC1A	0.967
ANAPC2	RBX1	0.959
CDC23	ANAPC11	0.959
ORC1	DBF4	0.958
CDK1	CCNB2	0.958
CDC23	RBX1	0.958
MCM2	CDC6	0.957
ANAPC11	BUB1B	0.956
CDC45	MCM5	0.956
CDK1	CCNA2	0.955
RAD21	STAG2	0.954
CDC45	ORC1	0.950
ORC1	CDC7	0.950
DBF4	MCM5	0.949
DBF4	CDC7	0.947
MCM5	CDC6	0.943
CDC7	MCM5	0.942
MCM7	CDC6	0.931
ORC1	MCM7	0.931
CDC27	ANAPC11	0.931
FZR1	BUB1B	0.928
FZR1	ANAPC11	0.926
TFDP1	RBL1	0.924
MCM6	CDC7	0.923
FZR1	MAD2L1	0.922
DBF4	MCM2	0.922
TFDP1	E2F2	0.922
MCM7	CDC7	0.921
CCNA2	CDC6	0.918
CHEK1	ATR	0.914
ATR	CCNB2	0.913

CDK1	CDC6	0.905
SMC1A	PLK1	0.892
SMC3	STAG2	0.891
ATR	CDC7	0.888
SMC1A	STAG2	0.884
CDC27	FZR1	0.880
ORC6	MCM5	0.876
CDC7	ORC6	0.868
DBF4	ORC6	0.868
CDC45	ORC6	0.868
ATR	ATM	0.864
ANAPC2	MAD2L1	0.859
ORC6	CDC6	0.855
ORC1	ORC6	0.852
DBF4	MCM4	0.848
MCM7	DBF4	0.848
CDC27	MAD2L1	0.835
CDC27	BUB3	0.833
YWHAG	CDC25B	0.833
YWHAB	CDC25B	0.833
MCM6	CDC6	0.813
SMC3	PLK1	0.807
MCM4	ORC6	0.792
MCM7	ORC6	0.792
BUB1B	RBX1	0.790
CDK1	ATR	0.788
CDK1	SKP2	0.786
WEE1	CDK1	0.782
CDK1	CDC25C	0.779
RBL1	E2F2	0.779
RBL1	E2F1	0.779
CDK1	CDC25A	0.779
CDK1	CDC25B	0.779
FZR1	CDC25A	0.779
CDK1	FZR1	0.779
CHEK2	PLK1	0.777
CDC27	PTTG1	0.774
ANAPC11	PTTG1	0.774

YWHAZ	YWHAG	0.774
SFN	YWHAG	0.774
YWHAZ	YWHAB	0.774
SKP2	CCNA2	0.774
SFN	YWHAZ	0.774
YWHAG	YWHAB	0.774
FZR1	PTTG1	0.774
PTTG1	ANAPC2	0.774
CDC23	BUB3	0.774
TFDP1	RBL2	0.773
CDK1	CCNE1	0.771
E2F1	RBL2	0.757

Supplementary Table S4. Significantly enriched KEGG pathway protein products (Top 25) from genes differentially expressed in HEC-1-B after VP treatment.

Pathway ID	Pathway description	Observed gene count	False Discovery Rate	Matching proteins in network (labels)
04110	Cell cycle	59	2.2e-135	ABL1, ANAPC11, ANAPC2, ATM, ATR, BUB1, BUB1B, BUB3, CCNA2, CCNB2, CCNE1, CCNE2, CDC14B, CDC23, CDC25A, CDC25B, CDC25C, CDC27, CDC45, CDC6, CDC7, CDK6, CDKN2A, CHEK1, CHEK2, DBF4, E2F1, E2F2, EP300, FZR1, GADD45B, HDAC2, MAD2L1, MCM2, MCM4, MCM5, MCM6, MCM7, ORC1, ORC6, PLK1, PRKDC, PTTG1, RAD21, RBL1, RBL2, RBX1, SFN, SKP2, SMC1A, SMC3, STAG2, TFDP1, TGFB1, TGFB2, WEE1, YWHAB, YWHAG, YWHAZ
04114	Oocyte meiosis	18	1.47e-25	ANAPC11, ANAPC2, BUB1, CCNB2, CCNE1, CCNE2, CDC23, CDC25C, CDC27, MAD2L1, PLK1, PTTG1, RBX1, SMC1A, SMC3, YWHAB, YWHAG, YWHAZ
05166	HTLV-I infection	19	2.84e-20	ANAPC11, ANAPC2, ATM, ATR, BUB1B, BUB3, CCNB2, CDC23, CDC27, CDKN2A, CHEK1, CHEK2, E2F1, E2F2, EP300, MAD2L1, PTTG1, TGFB1, TGFB2
04914	Progesterone-mediated oocyte maturation	13	4.83e-18	ANAPC11, ANAPC2, BUB1, CCNA2, CCNB2, CDC23, CDC25A, CDC25B, CDC25C, CDC27, FZR1, MAD2L1, PLK1
04115	p53 signaling pathway	11	2.85e-15	ATM, ATR, CCNB2, CCNE1, CCNE2, CDK6, CDKN2A, CHEK1, CHEK2, GADD45B, SFN
05203	Viral carcinogenesis	14	6.3e-15	CCNA2, CCNE1, CCNE2, CDK6, CDKN2A, CHEK1, EP300, HDAC2, RBL1, RBL2, SKP2, YWHAB, YWHAG, YWHAZ
05206	MicroRNAs in cancer	13	8.73e-15	ABL1, ATM, CCNE1, CCNE2, CDC25A, CDC25B, CDC25C, CDK6, CDKN2A, E2F1, E2F2, EP300, TGFB2
05161	Hepatitis B	11	8.83e-12	CCNA2, CCNE1, CCNE2, CDK6, E2F1, E2F2, EP300, TGFB1, TGFB2, YWHAB, YWHAZ
05200	Pathways in cancer	13	2.24e-10	ABL1, CCNE1, CCNE2, CDK6, CDKN2A, E2F1, E2F2, EP300, HDAC2, RBX1, SKP2, TGFB1, TGFB2
05220	Chronic myeloid leukemia	8	7.04e-10	ABL1, CDK6, CDKN2A, E2F1, E2F2, HDAC2, TGFB1, TGFB2
04068	Fox signaling pathway	9	1.82e-09	ATM, CCNB2, EP300, GADD45B, PLK1, RBL2, SKP2, TGFB1, TGFB2
05212	Pancreatic cancer	6	5.77e-07	CDK6, CDKN2A, E2F1, E2F2, TGFB1, TGFB2
03030	DNA replication	5	1.23e-06	MCM2, MCM4, MCM5, MCM6, MCM7
04350	TGF-beta signaling pathway	6	2.04e-06	EP300, RBL1, RBX1, TFDP1, TGFB1, TGFB2
04120	Ubiquitin mediated proteolysis	7	2.45e-06	ANAPC11, ANAPC2, CDC23, CDC27, FZR1, RBX1, SKP2
05222	Small cell lung cancer	6	3.01e-06	CCNE1, CCNE2, CDK6, E2F1, E2F2, SKP2
05169	Epstein-Barr virus infection	7	2.22e-05	CCNA2, EP300, HDAC2, SKP2, YWHAB, YWHAG, YWHAZ
05215	Prostate cancer	5	8e-05	CCNE1, CCNE2, E2F1, E2F2, EP300
05223	Non-small cell lung cancer	4	0.000286	CDK6, CDKN2A, E2F1, E2F2
05214	Glioma	4	0.000442	CDK6, CDKN2A, E2F1, E2F2
05211	Renal cell carcinoma	4	0.000509	EP300, RBX1, TGFB1, TGFB2
05218	Melanoma	4	0.000654	CDK6, CDKN2A, E2F1, E2F2
04151	PI3K-Akt signaling pathway	7	0.000692	CCNE1, CCNE2, CDK6, RBL2, YWHAB, YWHAG, YWHAZ
04390	Hippo signaling pathway	5	0.000823	TGFB1, TGFB2, YWHAB, YWHAG, YWHAZ
05219	Bladder cancer	3	0.00199	CDKN2A, E2F1, E2F2

Supplementary Table S5. Significantly enriched Pfam protein domains in HEC-1B after VP treatment:

Pathway ID	Pathway Description	Observed gene count	False Discovery Rate	Matching proteins in network (labels)
PF00244	14-3-3 protein	4	6.95e-06	SFN, YWHAB, YWHAG, YWHAZ
PF00493	MCM2/3/5 family	3	2.39e-05	MCM2, MCM5, MCM6
PF14551	MCM N-terminal domain	3	2.39e-05	MCM2, MCM5, MCM6
PF02463	RecF/RecN/SMC N terminal domain	2	0.0151	SMC1A, SMC3
PF06470	SMC proteins Flexible Hinge Domain	2	0.0151	SMC1A, SMC3

Table S6. Validation of RNASEQ data in HEC-1-B cell line.

Gene Description	Fold change after VP treatment	
	Fold change	p-value
HSPA6	1.76	0.027765
C2orf88	3.06	0.334911
CYR61	-79.73	0.087528
THBS1	-201.94	0.123748
ANKRD1	-17.99	0.149632
BUB1B	-1057.12	0.082369
CDC23	-25.09	0.082425
MAD2L1	-6.80	0.174852
BUB3	-12.87	0.095956
CDC27	-495.56	0.065866

Results are based on cDNA qRTPCR. Each value represents pooled value (3 different treatments were taken from each control and VP treated group and run in duplicate, so that n=6). HEC-1-B cells were treated with 10nM VP for 3h and control cells were treated with DMSO. Fold change calculations were based on values obtained as follows: VP sample /control sample. p values are based on 1-way ANOVA (control vs. VP treatments).

Table S7. Fold change of cell cycle genes in EMCA cell lines after verteporfin treatment.

Gene Description	HEC-1-A		HEC-1-B	
	Fold change	p-value	Fold change	p-value
CCRK	-474.22	0.091018	-2581.81	0.073007
CDK2	-3.15	0.107108	-5.88	0.058367
CCND1	-20.73	0.093982	-13.73	0.052903
CCNE1	-8.93	0.101753	-5.12	0.085084
E2F1	-103.17	0.077449	-210.35	0.052173

Results are based on cDNA qRTPCR. Each value represents pooled value (3 different treatments were taken from each control and VP treated group and run in duplicate, so that n=6). EMCA cells were treated with 10nM VP for 3h and control cells were treated with DMSO. Fold change calculations were based on values obtained as follows: VP sample /control sample. p values are based on 1-way ANOVA (control vs. VP treatments).

Table S8. Fold change of cell cycle genes in mouse subcutaneous tumors after verteporfin treatment.

Gene Description	Fold change after VP treatment	
	Fold change	p-value
CCRK	-166.26	0.073997
CDK2	-61.53	0.044884
CCND1	-7.99	0.772534
CCNE1	-243.62	0.003639
E2F1	-29.98	0.016485
BUB1B	-9.39	0.330252
CDC23	-100.45	0.012136

Results are based on cDNA qRTPCR. Each value represents pooled value (3 tumor samples were taken from each control and VP treated group and run in duplicate, so that n=6). Fold change calculations were based on values obtained as follows: VP sample /control sample. p values are based on 1-way ANOVA (control vs. VP treatments).

Supplementary Table S9A. Details of the primer sequences used in the study.

Human Primers

All the primer sequences are based on human gene sequences. The primers were designed using PrimerQuest tool and obtained from Integrated DNA Technologies, Inc. (IDT)

NM No.	Gene	Forward sequence (5'→3')	Reverse sequence (5'→3')
NM_001798	CDK2	CTTATGAGGCAGGTGAGAGATG	GACAAGGGTGACAGAGACAAA
NM_178432	CDK20	GCTGGGTCAATTCCTTCTCTAC	GCTCGACTGGATGTTCTCATAC
NM_053056	CCND1	CACACACACACACACAAACC	CCTCCCTTCAACACTTCCTAAA
NM_001238	CCNE1	CTGTACTGAGCTGGGCAAATA	GGTGCAACTTTGGAGGATAGA
NM_005225	E2F1	TGCAGAGCAGATGGTTATGG	CTCCAAGCCCTGTCAGAAAT
NM_004661	CDC23	GGTCAACAAACGGGACTACA	CGGCGTAAGCTCTCCAATAA
NM_001211	BUB1B	CAAAGGGAAGCCGAGCTATT	CACAAGTGTCTTCTGGGTTAGG
NM_002155	HSPA6	AAAGCCACTGCTGGAGATAC	CTCATCAGGGTTGATGCTCTT
NM_019065	NECAB2	CTCCGACAGAACCACATCAAA	CCTCTGTCTCCCAGAACTCATA
NM_001042519	C2orf88	AGGTACAGAGGGCTGGAATA	CTGCCTGCTTACAAGACAAATAC
NM_001554	CYR61	TCGCATCCTATACAACCCTTTAC	CAGTACTTGGGCCGGTATTT
NM_003246	THBS1	CTCAGACCGCATTGGAGATAC	CCATCTGTAGGCGGTGAAAT
NM_014391	ANKRD1	GAAGGCTGCTCTGGAGAATAAA	AGATCCGCGCCATACATAATC

NM_002358	MAD2L1	GGACTCACCTTGCTTGTAACT	CGGACTTCCTCAGAATTGGTAATA
NM_013366	ANAPC2	CGTCTGTATGGGTGCTTCTT	CGCCTTGATGGCAGAGATATAG
NM_004725	BUB3	CCGAGTGGCAGTTGAGTATTT	GCAAGCGTAGTCCCATCATTA
NM_001114091	CDC27	CTACACCGCAATGCAAATACC	GCTGCTGGTCCTCCTAATAAA
NM_001101	ACTB	GGAAATCGTGCGTGACATTAAG	TAGTCCGCCTAGAAGCATTTG
NM_002046	GAPDH	GATTCCACCCATGGCAAATTC	GTCATGAGTCCTTCCACGATAC
NM_006347	PPIH	CACCTTCCACAGGGTCATAAA	ACTCAAGAACACCACCAAGAA
NM_000291	PGK1	GATTACCTTGCCTGTTGACTTTG	AGTGTCTCCACCACCTATGA
NM_005566	LDHA	GCCTGTGCCATCAGTATCTT	TGCAGTTCGGGCTGTATTT

Supplementary Table S9B.

Mouse primers

All the primer sequences are based on mouse gene sequences. The primers were designed using PrimerQuest tool and obtained from Integrated DNA Technologies, Inc. (IDT).

NM No.	Gene	Forward sequence (5'→3')	Reverse sequence (5'→3')
XM_006517033	CDK20	TCAAAGCCAAGCACGTAGAG	CCAGGTCCGACAGCATAAAT
NM_183417	CDK2	CAAGCGGATTTCAGCCAAAG	GTAGCAGTCAGCACCAGAAA
NM_007631	CCND1	GGGAAGTCTTGAGAAGGAGTTT	GGTCGCACTGACCATCTATAAG
NM_007633	CCNE1	GGAGGTGTGCGAAGTCTATAAG	TCAACTCCAGTGAGGAGAAATG
NM_007891	E2F1	CCTGCAGAACAGATGGTCATAG	CCCAAAGTCACAGTCAAAGAGA
NM_009773	BUB1B	GAGCGTCTGAATGAGGACTATG	CAGGTCAACACTGTAGGAGAAG
NM_178347	CDC23	ACCTGTCTGGAGAGAAGAAGA	CTGGGAGCGTAAGCTGTAATAA
NM_007393	ACTB	GAGGTATCCTGACCCTGAAGTA	GCTCGAAGTCTAGAGCAACATAG
NM_010699	LDHA	GGCTTGTGCCATCAGTATCT	CCCGCCTAAGGTTCTTCATTAT
NM_028677	PPIH	CACCTTCCACAGGGTCATAAA	TCTCGATCTTCCTCATCACTAGA

*Since there is sequence homology between human and mouse genes in GAPDH and PGK1, we used the same primers.

Supplementary Table S10A: Table showing details of primary antibodies used.

Antigen	Type	Dilution	Manufacturer	Catalogue No.
CCRK	Mouse monoclonal	1:300	Santa Cruz	sc-517320
CDK2	Mouse monoclonal	1:300	Santa Cruz	sc-6248
Cyclin D1	Mouse monoclonal	1:300	Santa Cruz	sc-20044
β-actin	Mouse monoclonal	1:1000	Santa Cruz	sc-47778
YAP	Rabbit polyclonal	1:500	Novus Biologicals	NB110-58358
OCT4	Mouse monoclonal	1:300	Bio-Rad	VMA00234

Supplementary Table S10B: Table showing details of secondary antibodies used.

**Type**	**Dilution**	**Manufacturer**	Catalogue No.
Goat Anti-Rabbit IgG-HRP	1:5000	Boston Bioproducts	B-1215
Goat Anti-Mouse IgG-HRP	1:5000	Boston Bioproducts	B-1115
m-IgGκ BP-HRP	1:2000	Santa Cruz	sc-516102

Figure 4. Supplementary Information – Western Blots.

Verteporfin treated HEC-1-B mouse subcutaneous tumors

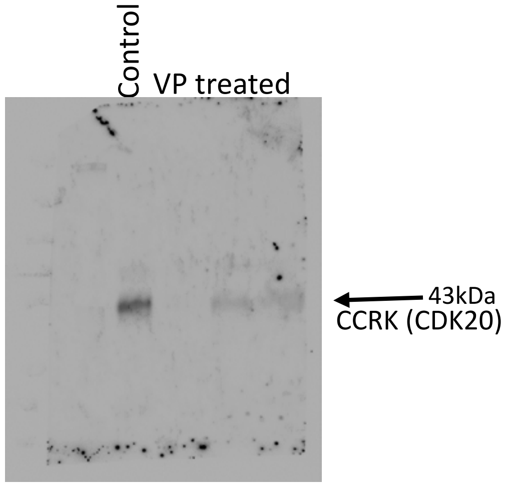

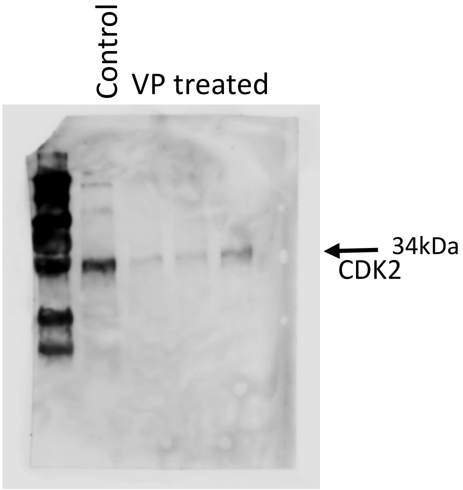

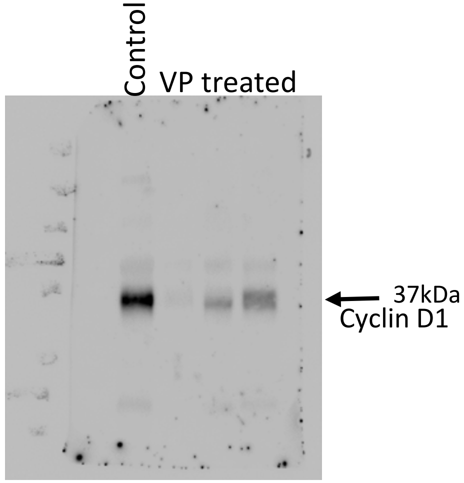

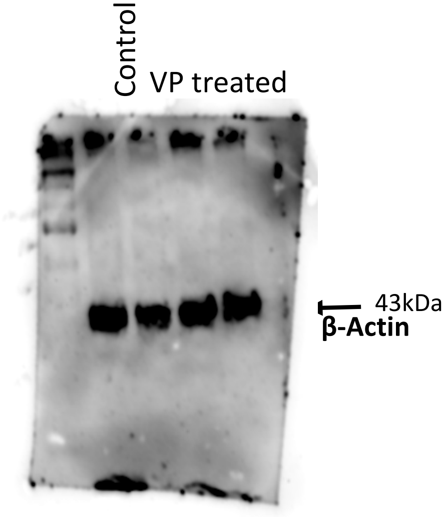

10% gels, each lane 40µg protein.
Blots are probed with the following:
All are anti-mouse antibodies.

Antibody	Primary	Secondary
CCRK	1:300	1:1000
CDK2	1:300	1:1000
Cyclin D1	1:300	1:1000
β-Actin	1:1000	1:5000

Supplementary Figure S5. Supplementary Information – Western Blots

A

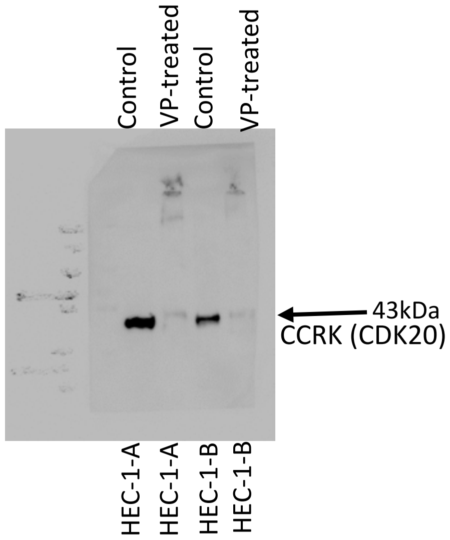

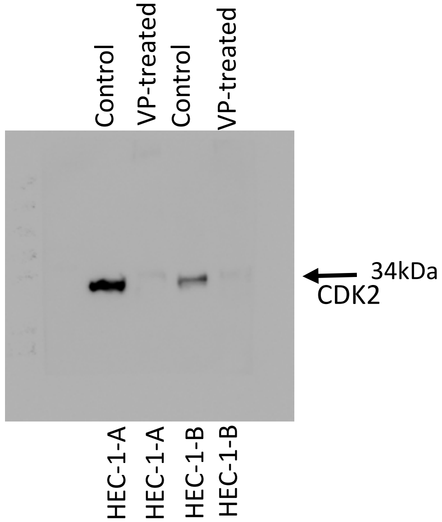

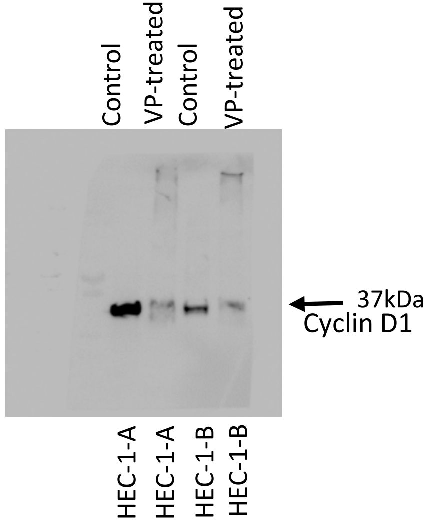

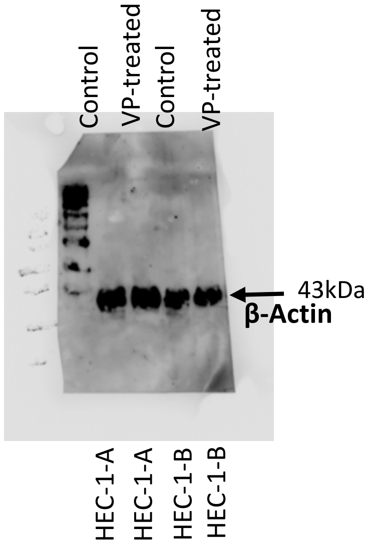

10% gels, each lane 40µg protein.
Blots are probed with the following:
All are anti-mouse antibodies.

Antibody	Primary	Secondary
CCRK	1:300	1:1000
CDK2	1:300	1:1000
Cyclin D1	1:300	1:1000
β-Actin	1:1000	1:5000

Figure S5. Supplementary Information – Western Blots.

B

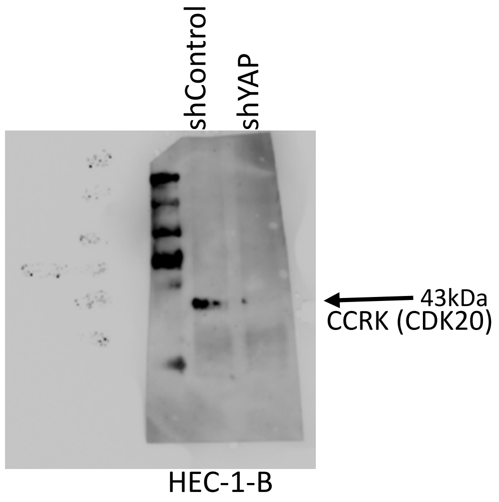

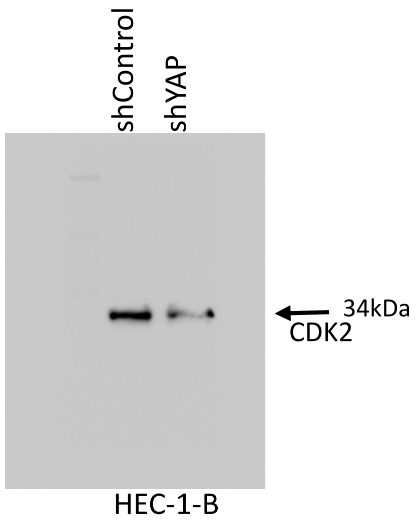

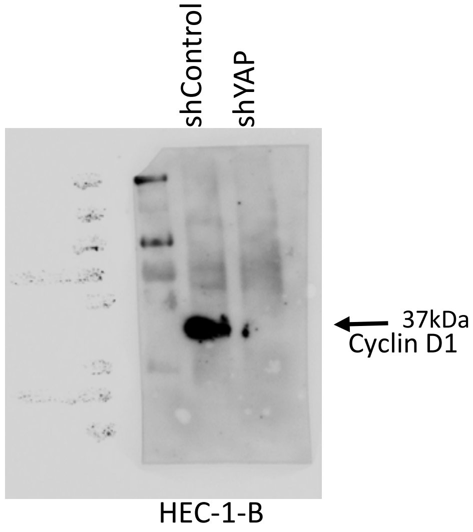

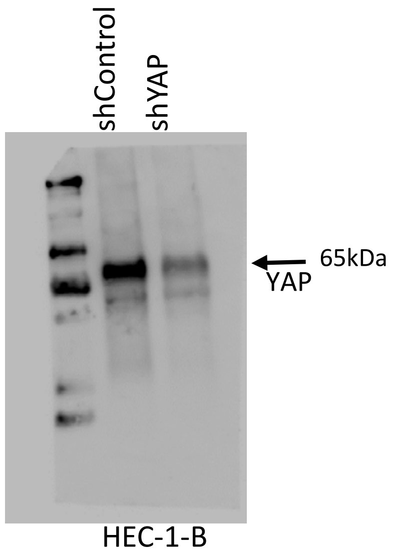

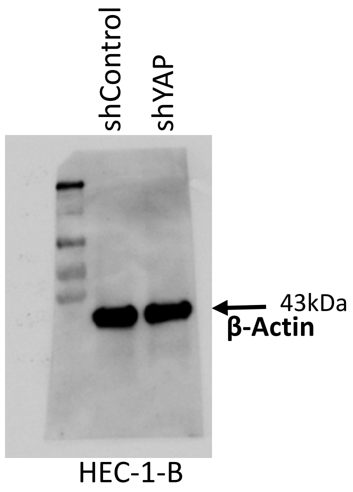

10% gels, each lane 40µg protein.
Blots are probed with the following:
All are anti-mouse antibodies, except YAP (anti-rabbit)

Antibody	Primary	Secondary
CCRK	1:300	1:1000
CDK2	1:300	1:1000
Cyclin D1	1:300	1:1000
YAP	1:500	1:1000
β-Actin	1:1000	1:5000

C

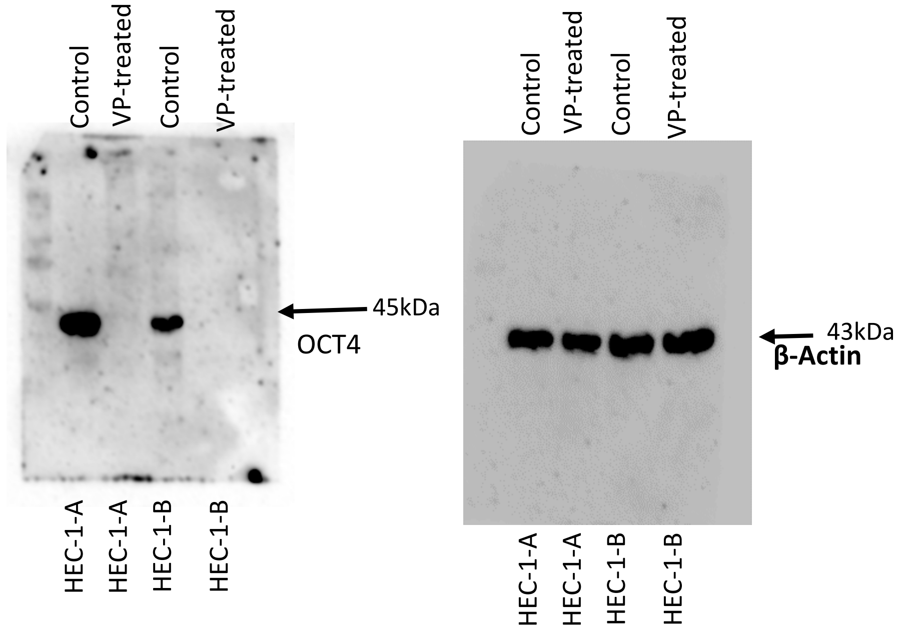

D

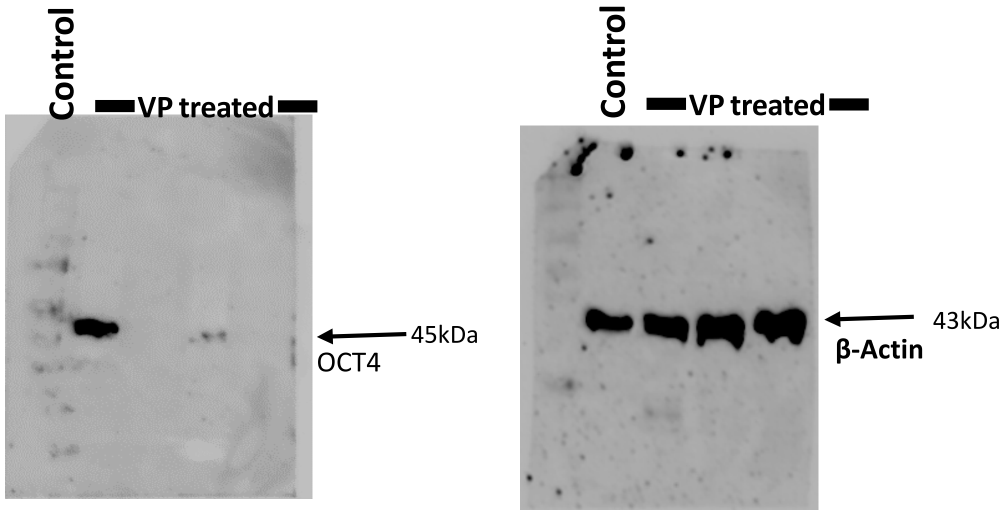

10% gels, each lane 40μg protein.
Blots are probed with the following:
All are anti-mouse antibodies.

Antibody	Primary	Secondary
OCT4	1:300	1:1000
β-Actin	1:1000	1:5000
